# Machine vision-based detection method for key phenotypic information of shiitake mushroom stipes

**DOI:** 10.3389/fpls.2025.1695008

**Published:** 2026-01-22

**Authors:** Jiuxiao Zhao, Mingfei Wang, Zuolin Li, Qiuxiao Song, Cheng Chen, Guoqiang Guo, Jing Dong, Feifei Shan, Ruixue Xu, Wei Liu, Xin Zhang, Wengang Zheng

**Affiliations:** 1 Intelligent Equipment Technology Research Center,Beijing Academy of Agriculture and Forestry Sciences, Beijing, China; 2 NongXin Science & Technology (Beijing) Co., Ltd., Beijing, China; 3 Information Technology Research Center, Beijing Academy of Agriculture and Forestry Sciences, Beijing, China; 4 Shandong QiHe Biological Technology Co., Ltd., Zibo, Shandong, China

**Keywords:** shiitake mushroom breeding, detection of stipe phenotype, centerline detection, edge detection, deep learning

## Abstract

**Introduction:**

In the field of shiitake mushroom breeding, the difficulty and inaccuracy in measuring key stipe traits severely limit improvements in breeding efficiency and product quality. This study aims to address this technical challenge.

**Methods:**

A key trait detection method for mushroom stipes based on integrated image processing technology was proposed: 1) Developed the ACmix-ADown-YOLOv11n stipe detection model; 2) Input the detection bounding box into the EfficientSAM network for precise segmentation,then used OpenCV-based techniques to calculate 12 key phenotypic features; 3) Constructed the CoTAttention-YOLOv11n-Ghost-pose algorithm to predict the stipe centerline.

**Results:**

The ACmix-ADown-YOLOv11n model achieved an AP of 93.7% and a detection speed of 23.97 ms; the CoTAttention-YOLOv11n-Ghost-pose algorithm achieved an AP of 97.2%, a recall rate of 96.1%, and a detection speed of 22.09 ms. For different stipe length categories, the R² between predicted and actual values was 0.989 (extremely short, RMSE=0.030), 0.992 (short, RMSE=0.023), 0.989 (middle, RMSE=0.028), and 0.978 (long, RMSE=0.043).

**Discussion:**

Experimental results confirm the effectiveness and reliability of the proposed method. This study provides an efficient and accurate approach for detecting key stipe traits, offering significant support for advancing intelligent shiitake mushroom breeding and enhancing cultivation quality.

## 1 Introduction

Shiitake mushroom stipes, a by-product of shiitake production and processing, are rich in nutrients such as proteins, amino acids, and carbohydrates. Moreover, the cellulose content in the stipes is significantly higher than that in the caps, making the stipes potentially valuable in terms of both nutritional and medicinal applications (da Silva Milhorini et al., 2022). In 2024, China’s shiitake mushroom production reached approximately 13,152,000 tons, accounting for over 90% of the global total (Qi et al., 2025). As an important component of shiitake mushrooms, the stipes are closely associated with key phenotypic traits (Xiang et al., 2021) and weight characteristics (Mesele et al., 2024), which are essential indicators of overall mushroom quality and play a crucial role in shiitake mushroom breeding. Therefore, the detection and analysis of key phenotypic traits in shiitake mushroom stipes hold significant research and application value.

The traditional method of phenotype acquisition relies on manual measurement, which is subjective and inefficient. The heavy workload and unreliable results when handling large-scale populations have constrained the advancement of breeding technology (Liu et al., 2017). With the rapid development of sensor technology, modern imaging, computer science, and engineering, research on crop phenotyping systems based on 3D reconstruction has received strong support (Yang et al., 2024). These systems often utilize depth cameras (Ran et al., 2025) and LiDAR (light detection and ranging) sensors (Chen et al., 2025) to capture point cloud data, from which phenotypic information can be extracted through segmentation and analysis. However, when 3D technology is applied to mushroom phenotype detection, it presents several limitations, including high cost, environmental sensitivity, complex data processing, limited accuracy, and operational complexity (Xu et al., 2025), which hinder its widespread application. In contrast, machine vision-based image analysis methods (Jindal and Mittal, 2025), which use cameras to capture two-dimensional images of mushroom stipe for phenotypic measurement, offer advantages such as quantitative analysis, high reproducibility, and ease of integration with other techniques at a lower cost.

Machine vision-based phenotype detection methods are generally categorized into two types: those based on traditional image processing algorithms and those based on deep learning algorithms (Xu et al., 2024). The basis of phenotypic detection is edge contour information. Among the traditional approaches, edge detection methods have been widely studied. For example, Yang et al. (2024) proposed an adaptive edge detection method that integrates a multi-scale nearest neighbor operator with grid segmentation. Using bilateral pixel information and grid divergence, this method enhances edge generation and achieves higher accuracy than conventional techniques. Additionally, AS and Gopalan (2022) introduced an improved edge detection method for cloth sample cropping based on an overall nested edge detection framework. Their method adopts a sequential process of edge detection, refinement, and smoothing, ensuring the extracted cloth contours are continuous, smooth, and detailed to meet cutting machine requirements and industrial production standards. To address the limitations of traditional welding edge detection algorithms—such as inaccurate extraction, discontinuity, and susceptibility to noise—Zou et al. (2024) proposed an improved Canny algorithm for weld pool edge detection in complex environments. Their method uses a hybrid filter (dark channel pre-demisting + bilateral filtering) instead of the original Gaussian filter for enhanced image denoising, calculates gradient magnitude and direction via a four-way Sobel operator, and employs an enhanced quadratic Otsu algorithm to determine the dual thresholds for edge extraction and connection. Experimental results demonstrate that this improved Canny algorithm can extract weld pool edge information more accurately and comprehensively than traditional methods. Ji et al. (2022) also proposed an improved Canny algorithm to overcome the limitations of Gaussian filter denoising and manually set thresholds in the traditional approach. Their method replaces Gaussian filtering with the Mean Shift algorithm, which better preserves edge information during noise reduction. Additionally, the maximum interclass variance algorithm is employed to determine an adaptive optimal threshold, thereby improving the algorithm’s adaptability. Verma et al. (2023) introduced an edge detection technique that combines edge-preserving bootstrap filtering with particle swarm optimization to address incorrect edge detection. In this method, the edge-preserving guided filter is applied during edge detection using the particle swarm optimization algorithm, effectively preserving edges while smoothing the background. In summary, traditional image processing methods are effective for detecting edge details; however, in this study, the target of detection is mushroom stipes which exhibit diverse morphologies and require algorithms with strong generalization capabilities for detecting key phenotypic indicators. Therefore, traditional edge detection algorithms have certain limitations in this context.

In recent years, with the advancement of deep learning techniques, deep learning-based edge detection methods have also been increasingly developed. Sahu et al. (2020) proposed the use of YOLO v3 for real-time license plate number recognition. Traditional approaches typically focus on contouring, segmentation, and edge detection processes but often suffer from low accuracy. This method offers improved accuracy for real-time license plate detection in India. Elharrouss et al. (2023) proposed a method to address complex target edge challenges to achieve more accurate edge detection. Preserving high-resolution edge details during training and maintaining resolution across network stages, the module connects to the previous layer’s output at each stage. An affine-parameter batch normalization layer is added after each layer as an erosion operation to enhance image uniform regions and improve algorithm accuracy. To estimate the quantity of banana fruit clusters during harvesting, Wu et al. (2023) proposed a deep learning-based identification method for entire banana fruit clusters. This method uses edge detection to extract individual fruit finger centroid points, applies clustering to identify the optimal number of visible bunches, and develops an estimation model based on bunch spiral arrangement to predict total bunches (including non-visible ones). Liu et al. (2022) proposed a deep learning model capable of intelligently distinguishing between meaningful and irrelevant edges, thereby generating high-quality edge maps with reduced noise. Furthermore, an arc growth-based ellipse detection method was introduced to fully exploit the advantages of these high-quality edge maps. Experimental results demonstrate that the proposed method significantly outperforms state-of-the-art approaches in terms of precision, recall, and F-score on real industrial images.

In summary, this study proposes a method for detecting key phenotypic traits of shiitake mushroom stipes by integrating image processing techniques with high-precision weight sensing technology. The aim is to achieve comprehensive phenotypic analysis of shiitake mushroom stipes. The specific research objectives are as follows:

1. To develop an improved YOLOv11-based object detection algorithm and input the detection results into EfficientSAM through bounding box prompting to perform image segmentation, thereby enabling accurate extraction of the edge contour information of mushroom stipes.
2. To design a phenotypic feature extraction algorithm based on OpenCV using the edge contour data, calculate key phenotypic parameters, and evaluate its performance under both red and green background conditions.
3. To innovatively enhance the pose estimation algorithm based on YOLOv11 by labeling the centerlines of mushroom stipes for training, thereby enabling accurate centerline detection of irregularly shaped stipes.

## 2 Materials and methods

### 2.1 Image acquisition device

**Figure 1:**
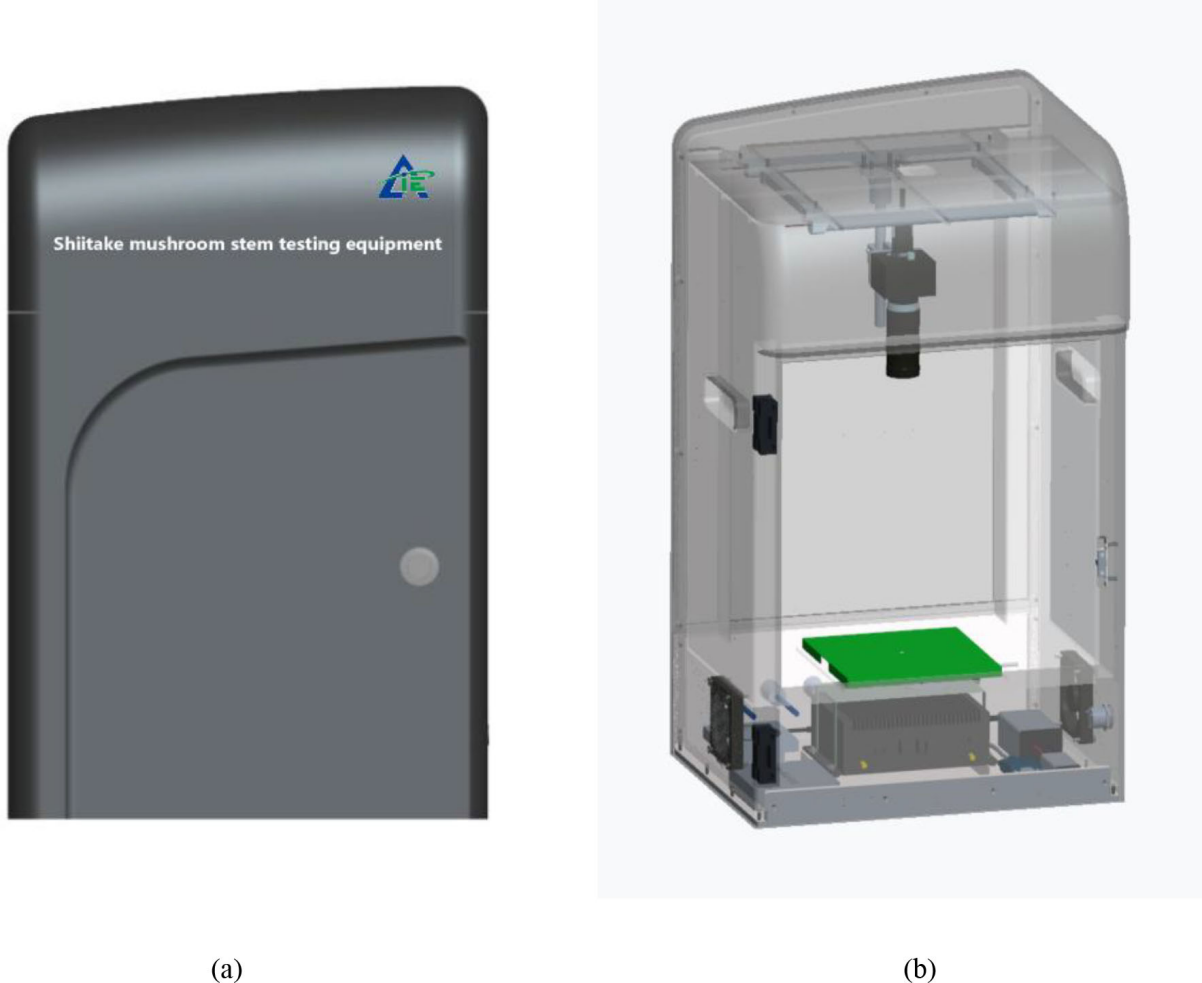
Diagram of the phenotypic apparatus of shiitake mushroom stipes **(a)** External view of the mushroom stipes phenotype detection equipment **(b)** Internal view of the mushroom stipes phenotype detection equipment.

The experimental site was located in the National Precision Agriculture Demonstration Base, Changping District, Beijing, with the coordinates of 116.46°E and 40.18°N. The appearance of the shiitake mushroom stipe phenotyping device is shown in **Figure 1a**; the frame structure of the device is shown in **Figure 1b**. The image acquisition device consists of an interface (USB interface between the camera and the host computer, model number: USB3-1-4-2-5M-Z), a case (customized, made of ABS(Acrylonitrile-butdiene-styrene) material, size 460mm×375mm×689mm), a camera (model number: MV-S2UC2000GM, 20 megapixels, resolution 5120×3840 pixels, pixel size 2.4μm×2.4μm, global exposure mode), a lens (fixed-focus lens MV-LD-16-20M-B, with medium pixel size, high resolution, and global exposure characteristics),red and green background plates, a fill light (120W four-color light source lamp, color temperature 5500K,set to 70% power during image acquisition), a USB extension port, Lenovo-branded industrial control computer, monitor, pressure sensor, high-precision weight sensor produced by Allison Technology (transmitted to the industrial control computer via a modbus converter),cooling fan and other components. The lens of the image acquisition equipment is 270 mm away from the background plate, and the field of view of the camera is 150 mm×150 mm, During image acquisition, the camera was set to JPEG format with an exposure time of 50ms and an ISO 100,which can acquire images of mushroom stipes more clearly.

### 2.2 Data acquisition

#### 2.2.1 Image data acquisition

**Figure 2:**
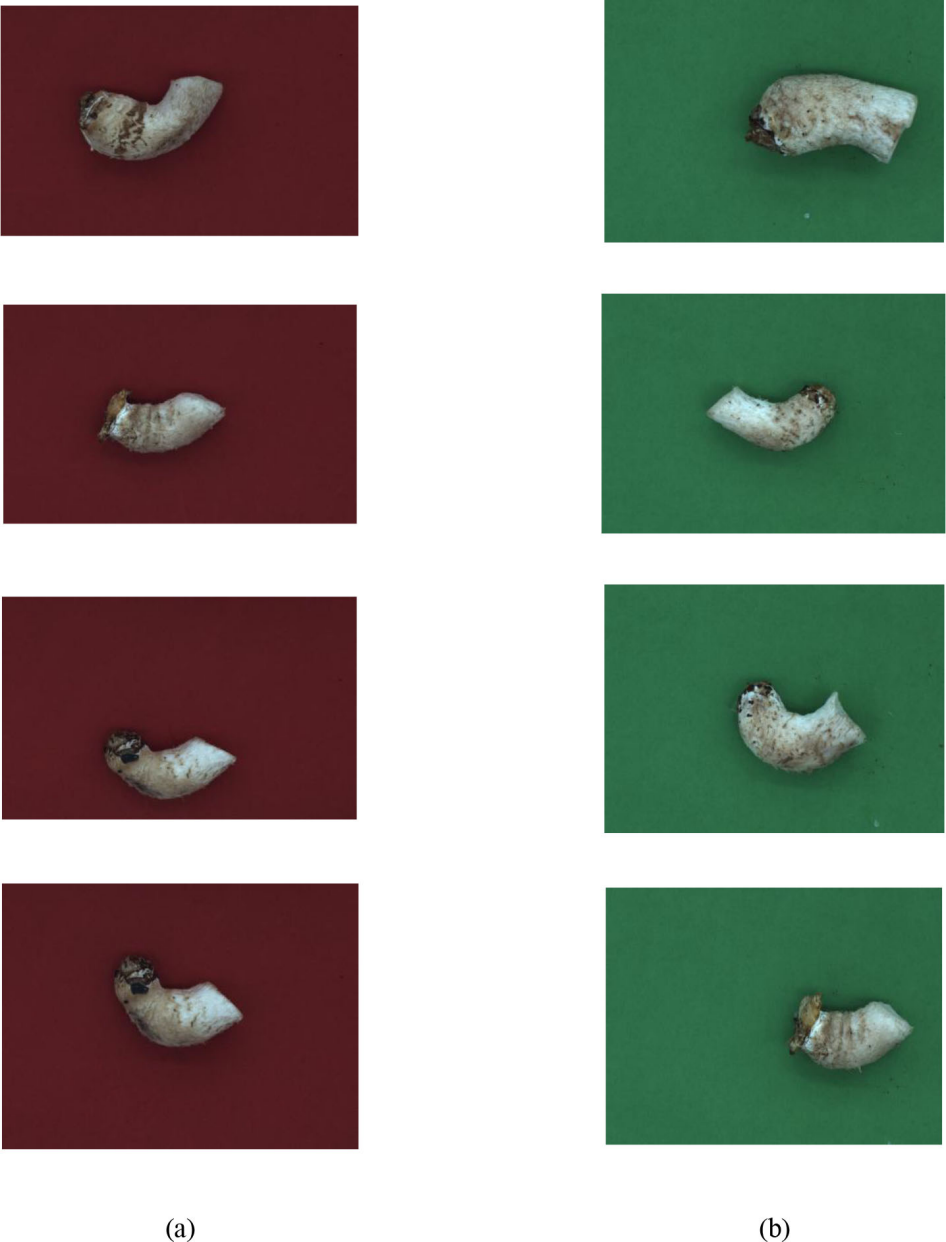
Top view of shiitake mushroom stipes under different background conditions **(a)** Red-shiitake mushroom stipes **(b)** Green-shiitake mushroom stipes.

**Figure 2** shows the pictures of shiitake stipes collected from July to November 2024 with red and green backgrounds. The data samples of shiitake mushroom stipes were provided by the Institute of Edible Mushroom Research, Shanghai Academy of Agricultural Sciences, and the variety was Shenxiang 1504; there were 985 samples in 8 batches. The image acquisition process was carried out in the acquisition device by setting the ROI for image acquisition and using a 13mm fixed focal length lens to take pictures of shiitake mushroom stipes under red and green backgrounds, respectively. The field of view of the camera lens was 150mm×150mm and 130mm×130mm after setting the ROI; the distance of the lens from the target was more than 200mm, and the image resolution was 5488×3672. 985 pictures of mushroom stipes with red and green backgrounds were collected respectively, totaling 1970 pictures. These images were divided into training, testing, and validation sets at a ratio of 7:2:1.

#### 2.2.2 Data on key traits of shiitake mushroom stipes

Referring to the DUS measurement standards for shiitake mushroom (Zheng et al., 2019), this study conducted detailed measurements and analyses on the species and key phenotypic traits of the collected shiitake mushroom stipes samples.

In this study, shiitake mushroom stipes samples were graded, and **Table 1** presents the grading statistics based on stipe length. The stipes were classified into five categories: extremely short, short, middle, long, and extremely long. There were 77 samples in the “extremely short” category, with lengths ranging from 0 to 11.56 mm; the “short” category contained the largest number of samples (731), with lengths between 11.57 and 27.69 mm; the “Middle” category included 141 samples with lengths ranging from 27.7 to 43.83 mm; and the “long” category consisted of 36 samples, with lengths from 43.84 to 59.96 mm. No samples were classified as “extremely long.” This classification will serve as a reference for the subsequent analysis of stipe phenotypic characteristics.

**Table 1:** Statistics on length grading of shiitake mushroom stipes.

Grading of shiitake mushroom stipes	Collection quantity	Minimum value (mm)	Maximum value (mm)
Extremely short	77	0	11.56
short	731	11.57	27.69
Middle	141	27.7	43.83
Long	36	43.84	59.96
Extremely long	0	59.97	—

**Table 2** lists the key trait indicators, measurement methods, units, and standards used in this study, which comprehensively cover all essential phenotypic traits of shiitake mushroom stipes. The measurement process strictly followed the specifications outlined in the table. Considering practical operability, outer rectangle length, outer rectangle width, maximum thickness, minimum thickness, average thickness, and weight were selected as measurable indicators. Among these, size-related indicators were measured in millimeters (mm), while weight was measured in grams (g). To ensure data accuracy and repeatability, each measurement was averaged across three independent trials. The color parameters of mushroom stipes—including the mean values of the red (R), green (G), and blue (B) color channels, as well as average grayscale intensity—were extracted from high-resolution images using MindVision image analysis software. Weight, as a critical indicator reflecting yield and growth conditions (Ma et al., 2024), was used to calibrate the pressure sensors within the 5g to 100g range, ensuring measurement reliability. Additional parameters, such as stipes centerline curvature, area, and perimeter, were extracted using machine vision-based image analysis algorithms.

**Table 2:** Measurement modalities for key trait indicators of stipes.

Key traits of the shiitake mushroom stipes	Measurement method	Organization	Standard
Outer rectangle length	Micrometer caliper	mm	Take an average of the results obtained from 3 measurements
Outer rectangle width	Micrometer caliper	mm	Take an average of the results obtained from 3 measurements
Centerline	—	—	—
Curvature	—	—	—
Area	—	mm²	—
Perimeter	—	mm	—
Maximum thickness	—	mm	Take an average of the results obtained from 3 measurements
Minimum thickness	—	mm	Take an average of the results obtained from 3 measurements
Average thickness	—	mm	Take an average of the results obtained from 3 measurements
R	—	—	—
G	—	—	—
B	—	—	—
Gray mean	—	—	—
Weight	Pressure sensor	g	5-100g weights

### 2.3 Method

**Figure 3:**
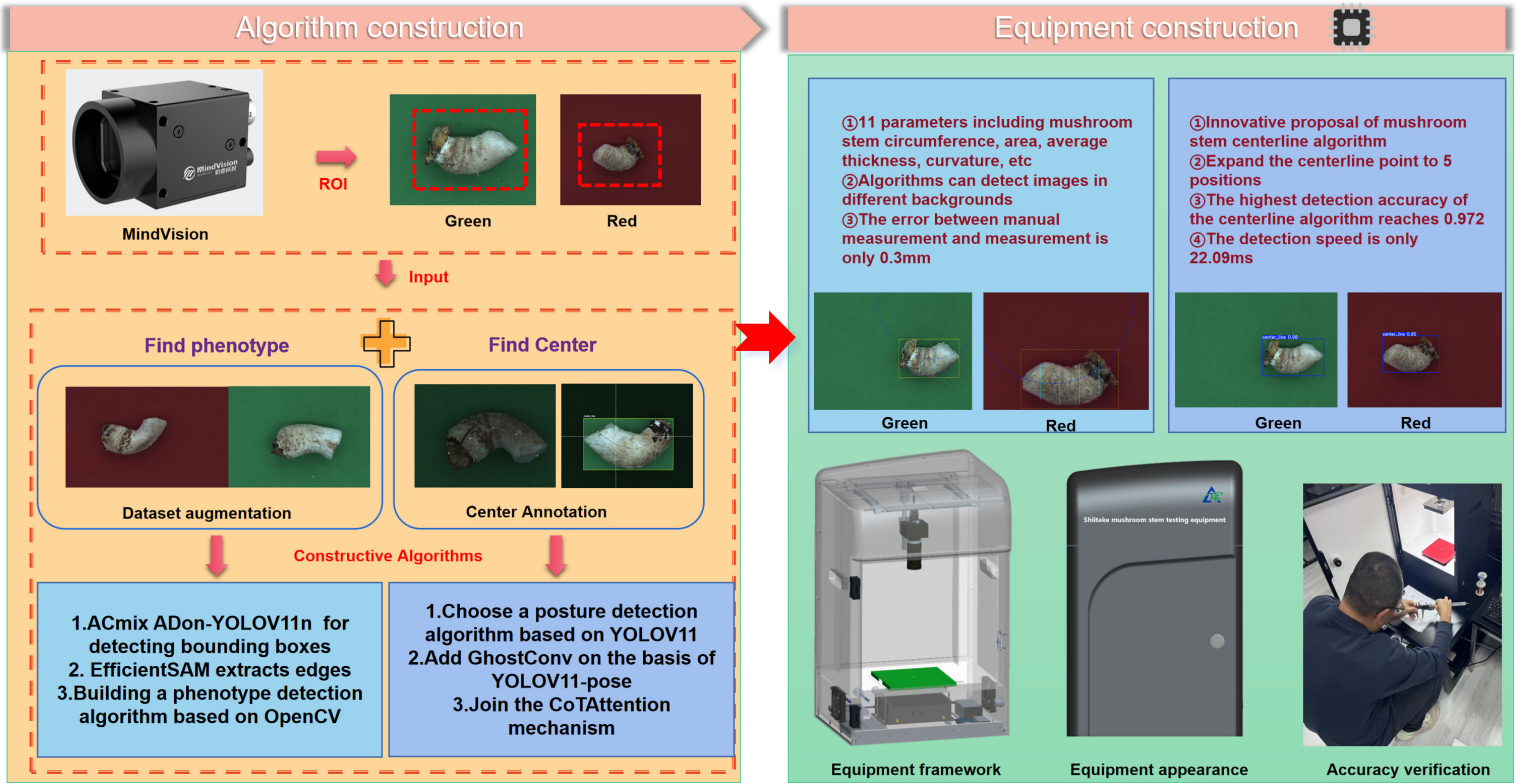
Overall model framework diagram.

This study aims to obtain the key phenotypic information of shiitake mushroom stipes and is composed of two main components, as illustrated in **Figure 3** The first component involves algorithm development, which begins with constructing a model based on a target detection network to rapidly acquire detection boxes and edge information following image segmentation. Subsequently, the key phenotypic traits of the stipes are extracted. Finally, a centerline recognition network is developed to capture the central features of the stipes. The second component focuses on deploying the phenotyping algorithm onto our phenotyping device, evaluating its performance, and conducting error analysis for validation. Specifically: (1) Focusing on the key traits of shiitake mushroom stipes, images are collected using phenotyping equipment, and the improved ACmix-ADown-YOLOv11 detection algorithm is employed to generate accurate stipe detection boxes; (2) Edge information acquisition is based on the precise and lightweight EfficientSAM model and a selected detection network, with a correlation established between the detection algorithm and the EfficientSAM model using the box tip as the core element; (3) The analysis of shiitake mushroom stipes traits relies on this accurate edge information, with algorithms from the OpenCV library utilized to construct a model for estimating key traits and validating the error between predicted and true values; (4) The centerline of the shiitake mushroom stipes is accurately extracted using CoTAttention-YOLOV11n-Ghost-pose as the foundation; (5) The performance of both the phenotype detection algorithm and the centerline algorithm is evaluated, and their accuracy is verified.

#### 2.3.1 ACmix-ADown-YOLOV11n algorithm

**Figure 4:**
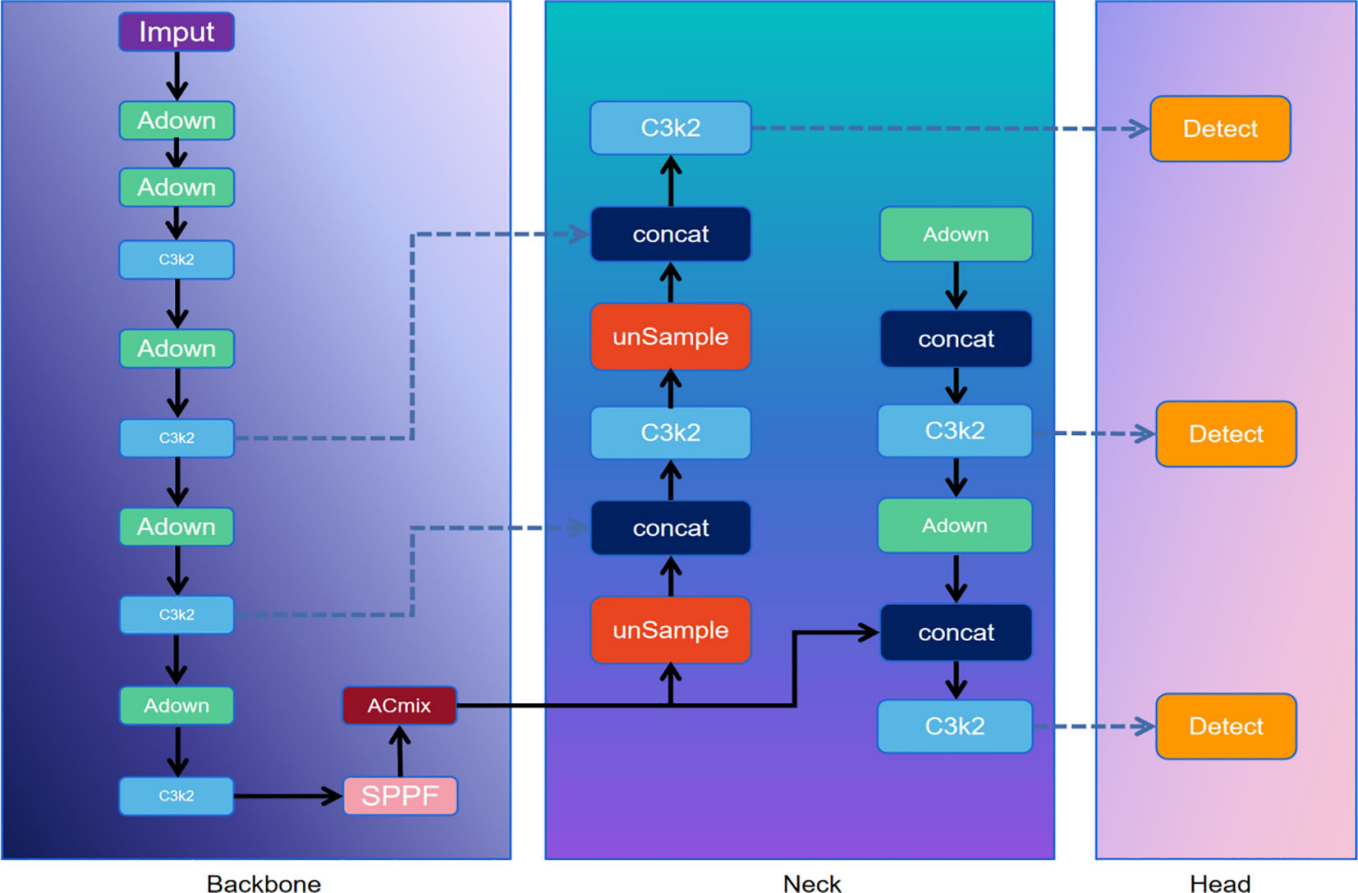
ACmix-ADown-YOLOV11n architecture.

The objective of this study is to identify the key phenotypic characteristics of mushroom stems. Since the detection frame outlining the mushroom stem contour serves only as a reference, a sufficiently accurate detection result is deemed adequate, and excessively high precision is not required. Therefore, this study adopts the lightweight YOLOv11n (He et al., 2025) as the baseline model, trained with training epochs=100, batch size=32, and learning rate=1e-4, using pycharm as the compiler and flask for algorithm packaging. To further enhance the model’s efficiency, the original convolutional layers are replaced with ADown (Zhang and Jiang, 2025). This approach effectively reduces the number of model parameters, thereby decreasing computational complexity, while preserving critical edge feature information of the mushroom stems to improve target identification accuracy. Additionally, the ACmix attention mechanism (Attention and Convolution Mixed) (Hao et al., 2025) is integrated into the model. This mechanism combines the global perception capability of self-attention with the local feature extraction ability of convolution, enabling a reduction in parameter count while maintaining model performance. The improved algorithm architecture is illustrated in **Figure 4**. Finally, the resulting detection frame is used as input for the box guidance module in EfficientSAM (Xiong et al., 2024).

##### 2.3.1.1 ACmix model

**Figure 5:**
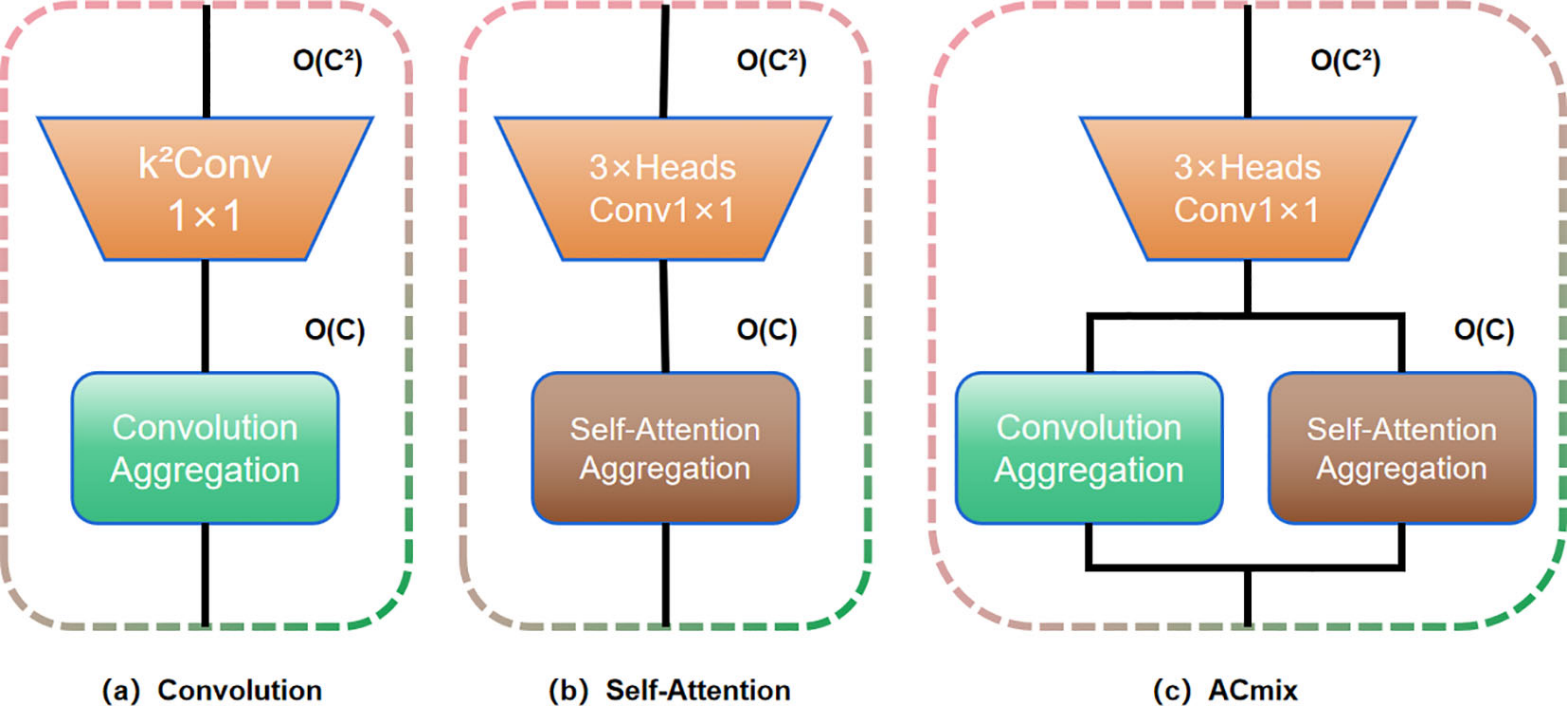
ACmix structure diagram. **(a)** Convolution; **(b)** Self-Attention; **(c)** ACmix.

The ACmix model demonstrates a substantial enhancement in both model performance and computational efficiency through the effective integration of convolution operations with the self-attention mechanism. The structural design of ACmix is presented in **Figure 5**. Its core innovation lies in utilizing 1×1 convolution to transform input feature maps into intermediate representations, followed by feature decomposition and fusion. This strategy enables both convolutional and self-attention components to share the same 1×1 convolution operation, thereby significantly reducing redundant computations. The dual-path architecture not only exploits the global contour perception capability offered by self-attention but also captures detailed local stipe contour features via convolution, all while maintaining a low computational footprint. The modular architecture of ACmix supports seamless integration into diverse network frameworks, thereby enhancing the network’s capacity for feature representation. Furthermore, ACmix improves computational efficiency and reduces model complexity by decomposing and reconstructing traditional convolutional and self-attention operations into more streamlined forms. This hybrid module design optimizes computational complexity across feature channels, enabling efficient feature extraction and improved model performance through shared computational resources and the synergistic combination of two complementary aggregation mechanisms. These improvements are achieved by integrating heterogeneous feature extraction strategies, ultimately contributing to enhanced model accuracy.

##### 2.3.1.2 ADown module

The ADown module is an innovative lightweight downsampling convolutional block designed to enhance the operational efficiency of YOLOv11n. Its core advantage lies in its compact architecture, which reduces model complexity by minimizing the number of parameters, thereby maintaining high computational efficiency even in lightweight models. While the primary function of ADown is to reduce the spatial resolution of shiitake mushroom stipes feature maps, it is also specifically designed to preserve critical image information, supporting more accurate contour detection. Additionally, the module incorporates adaptive learning capabilities that enable it to adjust for background variations in the dataset, thereby enhancing the generalization performance of the model.

In this study, the ADown module is flexibly integrated into both the backbone and detection head of YOLOv11n to replace conventional downsampling operations. Within the backbone, ADown effectively downsamples multiple layers of the feature map, while in the detection head, it further refines the resolution of the shiitake mushroom stipes feature maps. This dual integration strategy improves target detection accuracy while simultaneously reducing computational load.

#### 2.3.2 EfficientSAM segmentation module

EfficientSAM is a lightweight variant of the Segment Anything Model (SAM) that learns to reconstruct the features of the SAM image encoder through masked image modeling, enabling efficient visual representation learning. This approach not only reduces model complexity but also demonstrates superior performance across multiple vision tasks, including image classification, object detection, instance segmentation, and semantic segmentation. The EfficientSAM framework operates in two stages: pre-training of SAMI on the ImageNet dataset and fine-tuning of SAM on the SA-1B dataset. This two-stage strategy enables EfficientSAM to achieve performance comparable to the original SAM model while maintaining low computational costs. EfficientSAM incorporates a box cueing mechanism in instance segmentation tasks. This mechanism allows the model to accept a bounding box as input during inference, guiding it to perform detailed segmentation within the specified region. This feature is particularly beneficial for our shiitake mushroom stipes contour segmentation task, as it significantly narrows the processing area, thereby enhancing both segmentation efficiency and accuracy.

In this study, the output from the ACmix-ADown-YOLOv11n model is used as the box cue input for EfficientSAM. This cue guides the segmentation of shiitake mushroom stipes, enabling fast and precise boundary recognition and object segmentation. The resulting segmented image is then used to extract the maximum contour edges, which serve as the foundation for subsequent phenotypic trait analysis.

#### 2.3.3 Phenotypic characterization of shiitake mushroom stipes

The core objective of this study is to detect the phenotypic traits of shiitake mushroom stipes, which are categorized into basic phenotypic traits and complex phenotypic traits, such as centerline detection. Based on the accurate stipe edge information obtained through the previously proposed method, we are able to calculate 12 basic phenotypic features, including stipe outer rectangle length, outer rectangle width, curvature, and thickness, by implementing a phenotyping algorithm based on OpenCV. Due to variations in stipe pose, the centerline detection in this study is performed using an improved pose detection network based on YOLOv11-pose.

##### 2.3.3.1 Analysis of basic phenotypic information

In this study, the basic phenotypic indices were calculated using an OpenCV-based algorithm. First, the segmentation map generated by EfficientSAM was binarized, and contour detection was performed to extract the perimeter of the stipe. The cv2.contourArea function was used to compute the area enclosed by the contour, while cv2.boundingRect was applied to determine the outer rectangular bounding box, from which the outer rectangle length and width were derived. The cv2.bitwise_and function was employed to mask the original image using the segmentation mask, resulting in an image containing only the pixels within the stipe contour. For this extracted region, the cv2.calcHist function was used to calculate the RGB color histogram, providing information on the distribution of R, G, and B color channels, as well as the average grayscale intensity. To measure thickness, five equally spaced points were selected along the top and bottom edges of the outer rectangular box, and corresponding points were connected to form five cross-sectional lines. The intersection points between these lines and the stipe contour were identified, and pairs of these points were connected to calculate four thickness measurements. From these, the maximum, minimum, and average thickness values were computed.

For curvature analysis, the cv2.ximgproc.thinning function was used to extract the skeleton of the stipe. Due to the structural complexity of shiitake mushroom stipes, only the main skeleton branch was retained. The endpoints and midpoint of the main skeleton line were used to define two vertical lines, whose intersection served as the center of a fitted circle. The curvature was then defined as the inverse of the radius of this circle. Given the unique structure of the stipe, the centerline calculation method will be described in detail in a separate section.

##### 2.3.3.2 Shiitake mushroom stipe centerline detection

**Figure 6:**
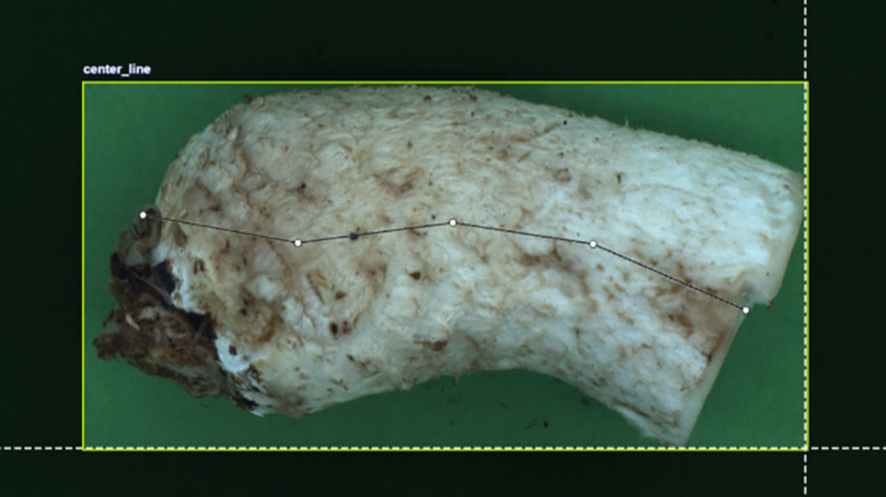
Shiitake mushroom stipes centerline labeling diagram.

To address the limitation that deep learning-based object detection typically outputs only candidate regions without extracting the centerline of the stipe, and that traditional OpenCV algorithms lack sufficient accuracy for centerline extraction, we propose an improved architecture. Specifically, we innovatively integrate the CoTAttention mechanism before the C2PSA layer in the backbone of the YOLOv11-pose pose estimation network and replace the standard convolutional layers in the detection head with GhostConv modules. The resulting CoTAttention-YOLOv11-pose-GHOST network is employed to predict the centerline of the shiitake mushroom stipes. During the data annotation phase, the centerline must be manually labeled. Due to the morphological variability of shiitake mushroom stipes, the positions of the two endpoints determine the length and direction of the centerline. While at least three coordinate points are theoretically required to define a curve, using too many points increases computational complexity without significantly improving accuracy. To balance accuracy and efficiency, this study selects five key points as the control points for centerline detection (as shown in **Figure 6**). The labeling rules for these five points are as follows: the leftmost and rightmost points represent the endpoints of the shiitake mushroom stipes, while the three intermediate points are evenly distributed along the length of the stipe, forming a quintuplet. Each key point is defined as the midpoint between the upper and lower edges of the corresponding position on the stipe.

For centerline detection, a total of 985 centerline images under red and green backgrounds were annotated in this study. These images were divided into training, testing, and validation sets at a ratio of 7:2:1. Specifically, 689 images were used for training, 197 for testing, and 99 for validation. These datasets were fed into the CoTAttention-YOLOv11-pose-GHOST pose detection network for model training.

##### 2.3.3.3 Conversion factors and errors

**Figure 7:**
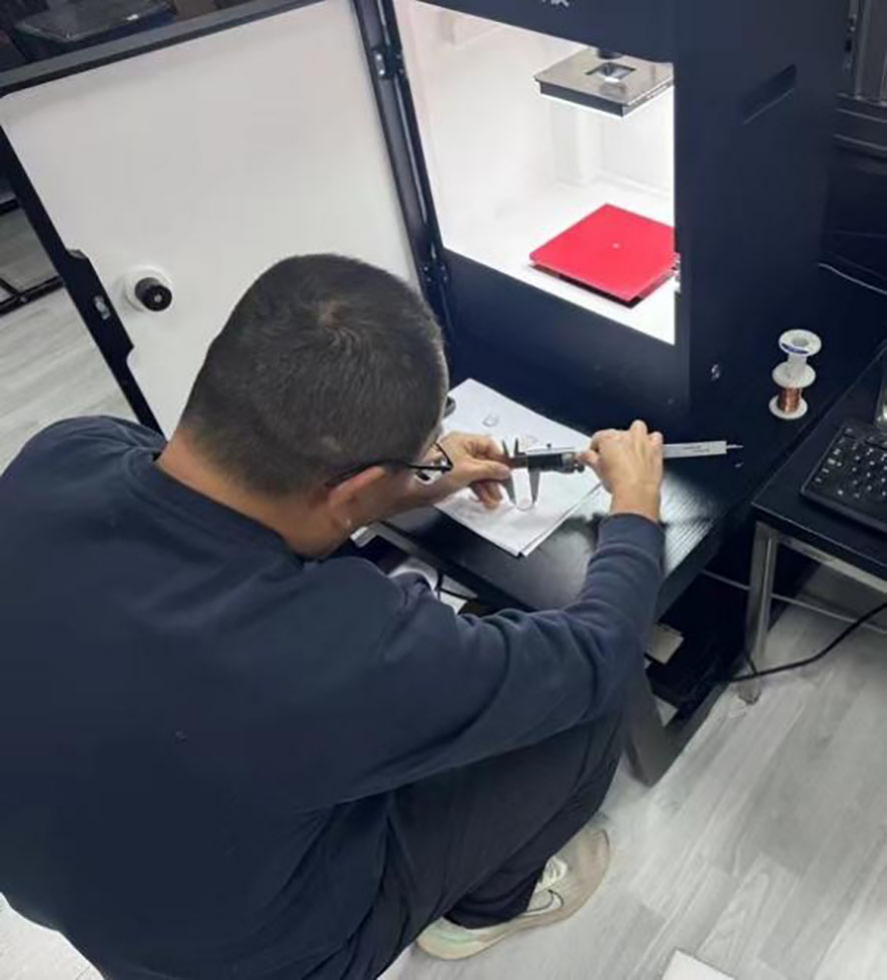
Conversion factor determination.

In this study, the camera lens was fixed at a distance of 270 mm from the background plate using a fixed bracket, with an image resolution of 5488 × 3672 pixels. Due to the soft and irregular texture of shiitake mushroom stipe, manual measurements using vernier calipers may introduce errors. Therefore, four types of standard reference objects were used to verify the system’s measurement accuracy (as shown in **Figure 7**): 1 Yuan RMB coin (diameter: 25.00 mm), 50-cent RMB coin (diameter: 20.50 mm), 10-cent RMB coin (diameter: 19.00 mm), and a 15 cm standard scale.

Firstly, line segments of 5 mm, 3 mm, and 1 mm were selected from the scale image, and the corresponding pixel counts were recorded. A pixel-to-real-world conversion coefficient was calculated using linear fitting, resulting in a value of 0.042 mm/pixel. Next, the width and height of the outer rectangles of the three types of coins were each measured three times using vernier calipers, and the average values were taken as the true values. Since the coins are round, the average of the width and height is equivalent to the diameter. Subsequently, the standard reference objects were placed within the field of view of the phenotyping equipment, and their positions were horizontally adjusted three times (each time at a different location). The width and height of the outer rectangles were measured using the image algorithm for each position, and the average of the three measurements was taken as the measured value. Finally, the absolute error between the true value and the measured value (true value – measured value) was calculated to evaluate the measurement accuracy of the system.

#### 2.3.4 Evaluation methods

In this study, the algorithms were evaluated using the following metrics: AP (Average Precision), Recall, mAP50, number of parameters, inference speed under PyTorch (measured in milliseconds), and model size. For centerline prediction, the evaluation metrics include Accuracy, Precision (Equation 1), Recall (Equation 2), Optimal Dataset Scale (ODS), per-image best threshold (OIS), and AP. The specific formulas are as follows:

(1) $$P\text{recision}=\frac{TP}{TP+FP}$$

(2) $$R\text{ecall}=\frac{TP}{TP+FN}$$

In order to evaluate the reliability of the algorithm for stipe phenotyping, this study used determination (R^2^) (Equation 3), root mean square error (RMSE) (Equation 4), mean square error (MSE) (Equation 5), and mean absolute error (MAE) (Equation 6) to assess the differences between the estimated and true values of the stipe phenotypic parameters. The differences between the estimated and true values of the phenotypic parameters of the stipe were evaluated.

(3) $$R^{2}=1-\frac{\sum_{i}{\left({\hat{y}}_{i}-y_{i}\right)}^{2}}{\sum_{i}{\left({\bar{y}}_{i}-y_{i}\right)}^{2}}$$

(4) $$RMSE=\sqrt{\frac{1}{m}\sum_{i=1}^{m}{\left(y_{i}-{\hat{y}}_{i}\right)}^{2}}$$

(5) $$MSE=\frac{1}{m}\sum_{i=1}^{m}(y_{i}-{\hat{y}}_{i}{)}^{2}$$

(6) $$MAE=\frac{1}{m}\sum_{i=1}^{m}|(y_{i}-{\hat{y}}_{i})|$$

Where ${\overset{ˆ}{\text{y}}}_{\text{i}}$ denotes the predicted value, ${\text{y}}_{\text{i}}$ represents the true value, and ${\bar{\text{y}}}_{\text{i}}$ indicates the average value. MAE reflects the actual error magnitude of the predicted values and is relatively insensitive to outliers. A smaller RMSE value indicates better model performance and reflects higher sensitivity to large errors. R² is a commonly used metric in regression analysis to evaluate the goodness of fit of the model, and the closer its value is to 1, the better the model fits the data.

## 3 Result

### 3.1 Phenotypic indicator performance

#### 3.1.1 Basic phenotypic indicator performance

We systematically evaluated the performance of various state-of-the-art lightweight target detection models on a specific dataset of shiitake mushroom stipes, including YOLOv5 (Deng et al., 2025), YOLOv6s (Demirel et al., 2025), YOLOv8-Ghost-p2 (Di et al., 2025), YOLOv9s (Gui et al., 2025), YOLOv10n (Liao et al., 2025), and YOLOv11n along with their improved versions, with the aim of identifying models that demonstrate excellent performance for stipe contour detection. **Table 3** details the key performance metrics of each model after 100 training epochs on the red and green datasets, covering Recall, Average Precision (AP), mean Average Precision at IoU=0.5 (mAP50), Giga Floating-Point Operations (GFLOPs), number of model parameters, and inference speed in the PyTorch framework. The analysis of recall reveals the outstanding performance of the ACmix-ADown-YOLOV11n model, which ranks first among all models with a value of 0.898 on a green background; this result indicates its high sensitivity in recognizing shiitake mushroom stipes. Comparatively, the ADown-YOLOV11n model has the lowest recall at 0.479 in the green background due to its oversimplified network structure; although it reaches a maximum detection speed of 15.89 ms, this still affects its comprehensiveness in target detection tasks. In evaluating AP scores, the ACmix-ADown-YOLOV11n model again demonstrates superiority on a green background with a score of 0.937 while achieving an AP score of 0.903 on a red background—metrics that reflect its ability to maintain high detection accuracy across different contexts. Although the YOLOv5s model shows acceptable performance with an AP score of 0.806 on a green background, its low recall (0.556) limits effectiveness in real-world applications. The mAP50 metric further consolidates ACmix-ADown-YOLOV11n’s leading position with an achievement score of 0.910 on a green background significantly outperforming other models; this result highlights both accuracy and reliability when processing shiitake mushroom stipes data—with C3Ghost-GhostConv-YOLOV11 achieving an mAP50 score of 0.820 also under similar conditions.

**Table 3:** Comparison of the performance of each edge detection network in different background.

Models	Epoch	Train on	Recall	AP	mAP50	GFLOPs	Parameters	Speed_PyTorch(ms)
YOLOV5s	100	Shiitake mushroom stipes(Red)	0.541	0.791	0.605	24.21	9153152	45.62
YOLOV6s	100	Shiitake mushroom stipes(Red)	0.517	0.805	0.636	44.87	16452192	36.01
YOLOV8-Ghost-p2	100	Shiitake mushroom stipes(Red)	0.602	0.741	0.608	8.76	1606492	29.55
YOLOV9s	100	Shiitake mushroom stipes(Red)	0.589	0.851	0.603	27.39	7287795	53.38
YOLOV10n	100	Shiitake mushroom stipes(Red)	0.506	0.807	0.591	8.39	2707430	21.98
YOLOV11n	100	Shiitake mushroom stipes(Red)	0.706	0.838	0.751	6.44	2590035	27.34
ADown-YOLOV11n	100	Shiitake mushroom stipes(Red)	0.469	0.838	0.434	5.26	2116299	16.66
DAT-Ghost-YOLOV11n	100	Shiitake mushroom stipes(Red)	0.607	0.836	0.665	5.79	2230475	20.86
C3Ghost-Ghostconv-YOLOV11n	100	Shiitake mushroom stipes(Red)	0.701	0.841	0.802	5.88	2293227	26.53
ACmix-ADown-YOLOV11n(ours)	100	Shiitake mushroom stipes(Red)	0.859	0.903	0.898	5.26	2116299	24.49
YOLOV5s	100	Shiitake mushroom stipes(Green)	0.556	0.806	0.701	24.21	9153152	45.23
YOLOV6s	100	Shiitake mushroom stipes(Green)	0.531	0.831	0.689	44.87	16452192	35.64
YOLOV8-Ghost-p2	100	Shiitake mushroom stipes(Green)	0.617	0.760	0.656	8.76	1606492	29.33
YOLOV9s	100	Shiitake mushroom stipes(Green)	0.625	0.884	0.687	27.39	7287795	53.2
YOLOV10n	100	Shiitake mushroom stipes(Green)	0.515	0.884	0.637	8.39	2707430	21.07
YOLOV11n	100	Shiitake mushroom stipes(Green)	0.753	0.892	0.806	6.44	2590035	26.99
ADown-YOLOV11n	100	Shiitake mushroom stipes(Green)	0.479	0.856	0.459	5.26	2116299	15.89
DAT-Ghost-YOLOV11n	100	Shiitake mushroom stipes(Green)	0.620	0.897	0.688	5.79	2230475	20.07
C3Ghost-Ghostconv-YOLOV11n	100	Shiitake mushroom stipes(Green)	0.745	0.854	0.820	5.88	2293227	25.79
ACmix-ADown-YOLOV11n(ours)	100	Shiitake mushroom stipes(Green)	0.898	0.937	0.910	5.26	2116299	23.97

In terms of computational efficiency, the GFLOPs of ACmix-ADown-YOLOv11n is 5.26, which is comparable to that of YOLOv5s. However, its parameter count is slightly higher at 2,116,299. Despite this increase in parameters, the inference speed of ACmix-ADown-YOLOv11n reaches a maximum of 23.97 ms, indicating that the model can maintain a fast processing speed while effectively managing computational complexity. This characteristic is particularly important for breeding scenarios that require real-time feedback of phenotypic data. Although YOLOv11n has a speed advantage, it performs poorly in terms of recall and average precision. This limitation primarily stipe from its simplified network structure, which sacrifices detection accuracy to improve inference speed. Additionally, the performance of YOLOv8-Ghost-p2 on AP and mAP50 metrics in the green background is mediocre, achieving scores of 0.760 and 0.656, respectively. This underperformance is attributed to the inability of its network design to adequately adapt to the specific characteristics of shiitake mushroom stipes.

In summary, the ACmix-ADown-YOLOV11n model achieves a fast processing speed while maintaining a high detection accuracy, which makes it a significant application potential in the field of real-time target detection. Its balanced performance in different performance metrics, especially its outstanding achievements in recall and average precision, proves its robustness and effectiveness in handling red and green datasets.

#### 3.1.2 Qualitative analysis of edge detection models

**Figure 8:**
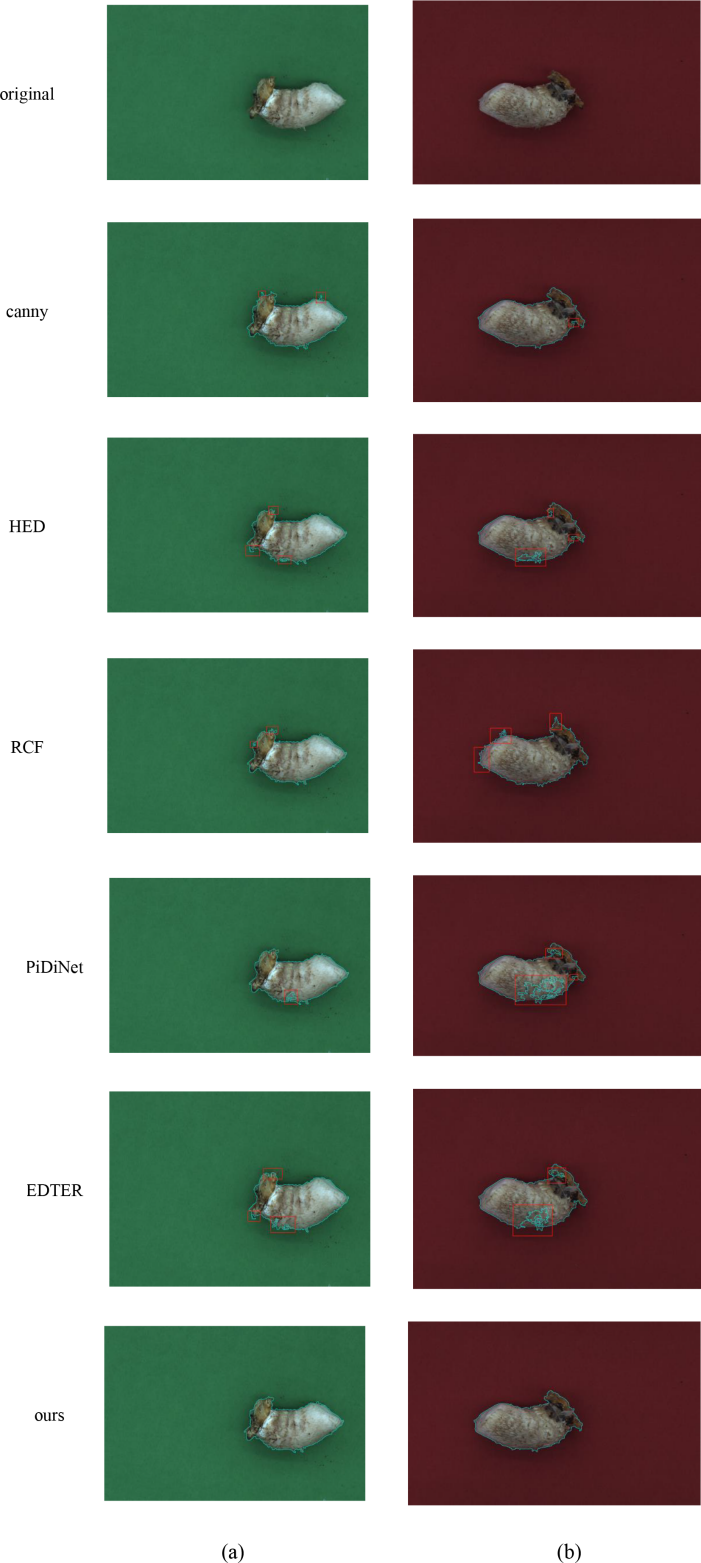
Effect of different edge detection algorithms on the edge recognition of shiitake mushroom stipes **(a)** Green-shiitake mushroom stipes **(b)** Red-shiitake mushroom stipes.

In order to evaluate the robustness and accuracy of stipe edge detection under varying background conditions and to support the extraction of fundamental phenotypic parameters—such as area, perimeter, average thickness, curvature, and color information—in the phenotypic analysis of shiitake mushroom breeding, this study employed both green and red backgrounds, as illustrated in **Figure 8**. Notably, in **Figure 8**, cyan represents the stipe edge information. The performance of several edge detection methods—Canny (a classical edge operator, Ji et al., 2025), HED (Miraldo et al., 2025), RCF (Wang et al., 2025), PiDiNet (Zhang et al., 2025), and DGCFN (Yang and Fang, 2025)—was compared with that of the proposed method, which is based on ACmix-ADown-YOLOv11n followed by edge extraction using EfficientSAM segmentation. Experimental results, reveal that existing methods generally suffer from edge detection deficiencies. Specifically, Canny relies on manually set gradient thresholds. Although gradient fluctuations caused by stipe texture are minimal in the red background, manual threshold adjustment leads to poor contour continuity due to the removal of weak edges. In the green background, contour fragmentation is more severe, indicating a lack of robustness to weak edges and texture disturbances. RCF exhibits poor performance due to limited feature representation. In the green background, the brown region at the top of the stipe shows low contrast, resulting in edge discontinuities (response missing). Additionally, in the red background, color similarity leads to misdetection of non-target regions (marked by red box). HED, despite multi-scale feature fusion, fails to adequately capture low-contrast edges. In the green background, the edge of the brown region at the top of the stipe is missing, while in the red background, surface texture triggers the generation of pseudo-edges (marked by red box). PiDiNet, due to its bias toward local detail learning and lack of global contour constraints, over-responds to texture in the red background (marked by red box) and experiences localized pseudo-edge flooding in the green background. Lastly, EDTER exhibits incomplete contour fitting (marked by red box).

In contrast, our approach achieves a tight fit in both backgrounds through three key components: deep learning feature extraction, adversarial background suppression, and end-to-end contour closure constraints. Specifically, deep learning feature extraction focuses on the trend of the stipe contour and local edge details to avoid breakage. Adversarial background suppression employs background discriminative branching, which explicitly distinguishes between target edges and background/internal texture, thereby reducing color and texture interference. End-to-end contour closure constraints enforce the output of continuous, closed contours that align with the stipe’s morphological features. As a result, contour integrity and anti-interference capability are enhanced by accurately capturing the strong contrast boundary between the stipe and the background on the green background, while effectively suppressing internal texture interference on the red background.

**Table 4** compares the performance of various edge detection algorithms on both red and green backgrounds in the task of detecting shiitake mushroom stipes edges, using ODS, OIS, and AP metrics. The traditional Canny algorithm does not require a training set and relies on manually designed filtering and thresholding rules for edge extraction. On the green background, it achieves an ODS of 0.881, an OIS of 0.878, and an AP of 0.923; on the red background, it achieves an ODS of 0.896, an OIS of 0.868, and an AP of 0.897, indicating a generally acceptable performance. Although deep learning algorithms such as HED, RCF, PiDiNet, and EDTER can theoretically learn features automatically through neural networks, most of them perform worse than Canny in this task. For example, HED achieves an ODS of only 0.797 on the green background, while RCF and PiDiNet achieve ODS and AP values mostly in the range of 0.7–0.8 on the red background. It can be observed that, except for our proposed algorithm, deep learning methods do not demonstrate a clear advantage in this specific task due to limitations in model design and training adaptation. Only our algorithm achieves superior accuracy, surpassing traditional methods. This result not only highlights the effectiveness of traditional algorithms in certain agricultural scenarios but also indicates the need for further improvements in deep learning models to better adapt to such environments.

**Table 4:** Performance comparison of different edge detection networks.

Models	Pub.’Year	Train on	ODS	OIS	AP
Canny	PAMI’86	/	.881	.878	.923
HED	ICCV’15	Green-shiitake mushroom stipes	.797	.786	.801
RCF	CVPR’17	/	.756	.785	.761
PiDiNet	CVPR’19	Green-shiitake mushroom stipes	.713	.728	.732
EDTER	CVPR’22	Green-shiitake mushroom stipes	.879	.873	.891
ours	—	Green-shiitake mushroom stipes	.971	.958	.983
Canny	PAMI’86	/	.896	.868	.897
HED	ICCV’15	/	.861	.864	.865
RCF	CVPR’17	Red-shiitake mushroom stipes	.736	.757	.734
PiDiNet	CVPR’19	Red-shiitake mushroom stipes	.703	.704	.718
EDTER	CVPR’22	Red-shiitake mushroom stipes	.861	.864	.865
ours	—	Red-shiitake mushroom stipes	.951	.935	.964

**Figure 9:**
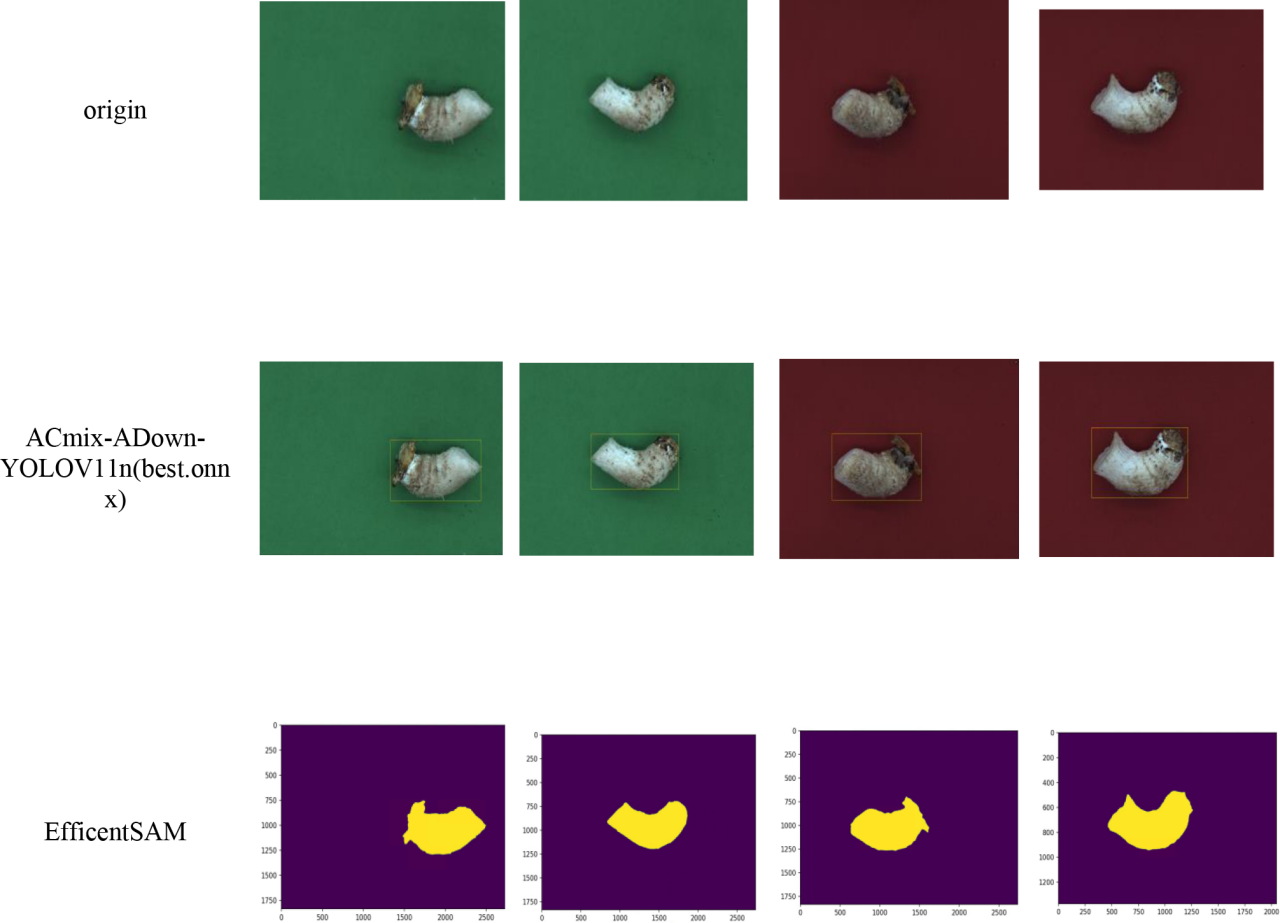
Process of basic phenotype detection of shiitake mushroom stipes.

**Figure 10:**
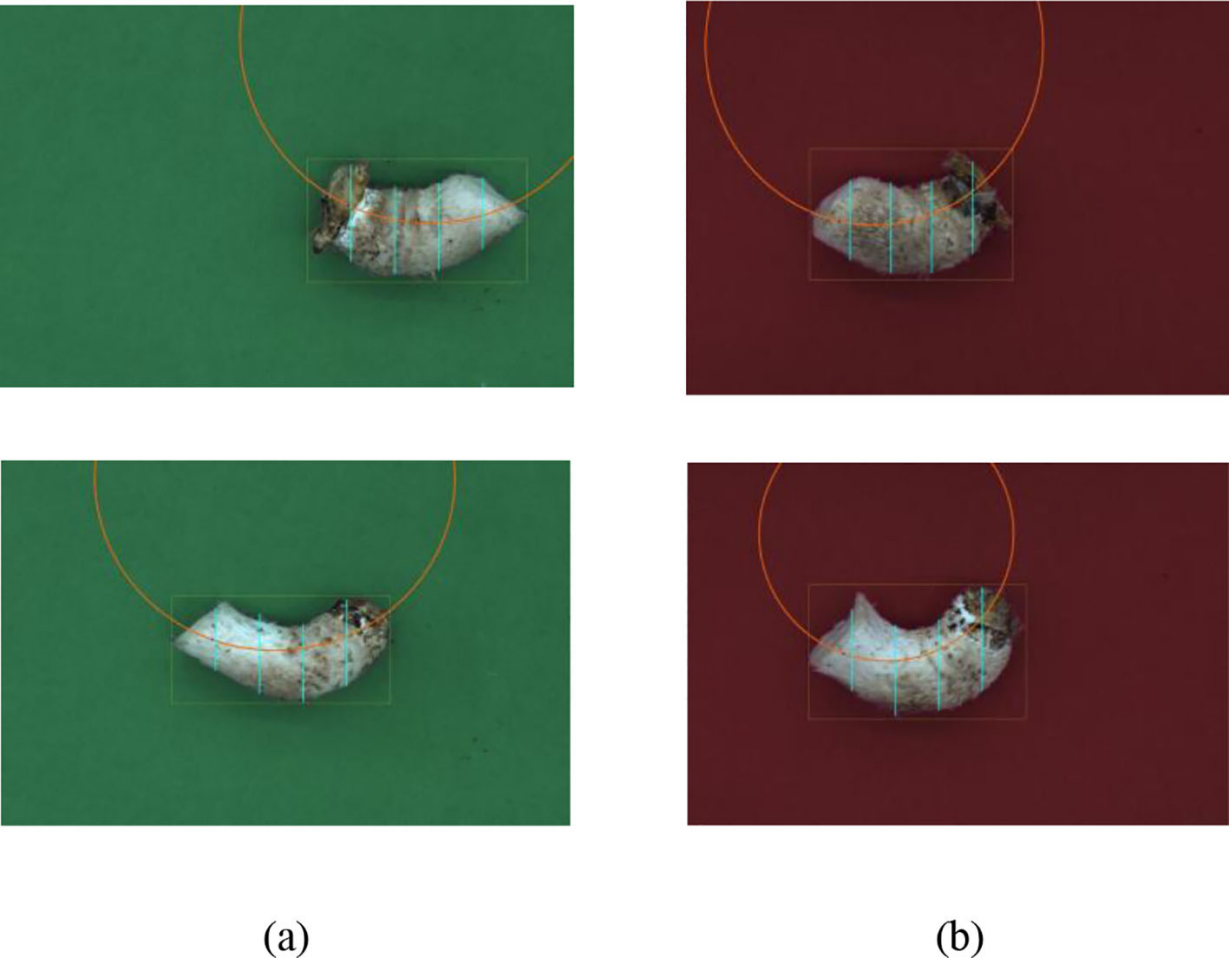
Basic phenotyping results **(a)** Green-shiitake mushroom stipes **(b)** Red-shiitake mushroom stipes.

In this study, our algorithm was able to obtain accurate stipe detection frames despite the specificity of fluffy interference at the stipe edge. After feeding the detection frame into the EfficientSAM segmentation network, it can be seen that the contour display is more complete under both red and green backgrounds, which is suitable for phenotype detection. Finally, we obtained 11 basic phenotypic indexes based on the stipe edges, and the overall process is shown in **Figures 9** and **10**.

#### 3.1.3 Performance of centerline detection model

In terms of shiitake mushroom stipes centerline detection, YOLOv8s-pose, YOLOv11n-pose, YOLOv11s-pose and the improved models (CoTAttention-YOLOv11n-pose, CoTAttention-YOLOv11n-pose-GHOST) are selected as experimental objects, and green and shiitake mushroom stipes images under red background are used as the dataset, and the number of training rounds is set to 100, and the performance is evaluated in terms of recall, AP, GFLOPs, Parameters, and PyTorch inference speed dimensions.

**Figure 11:**
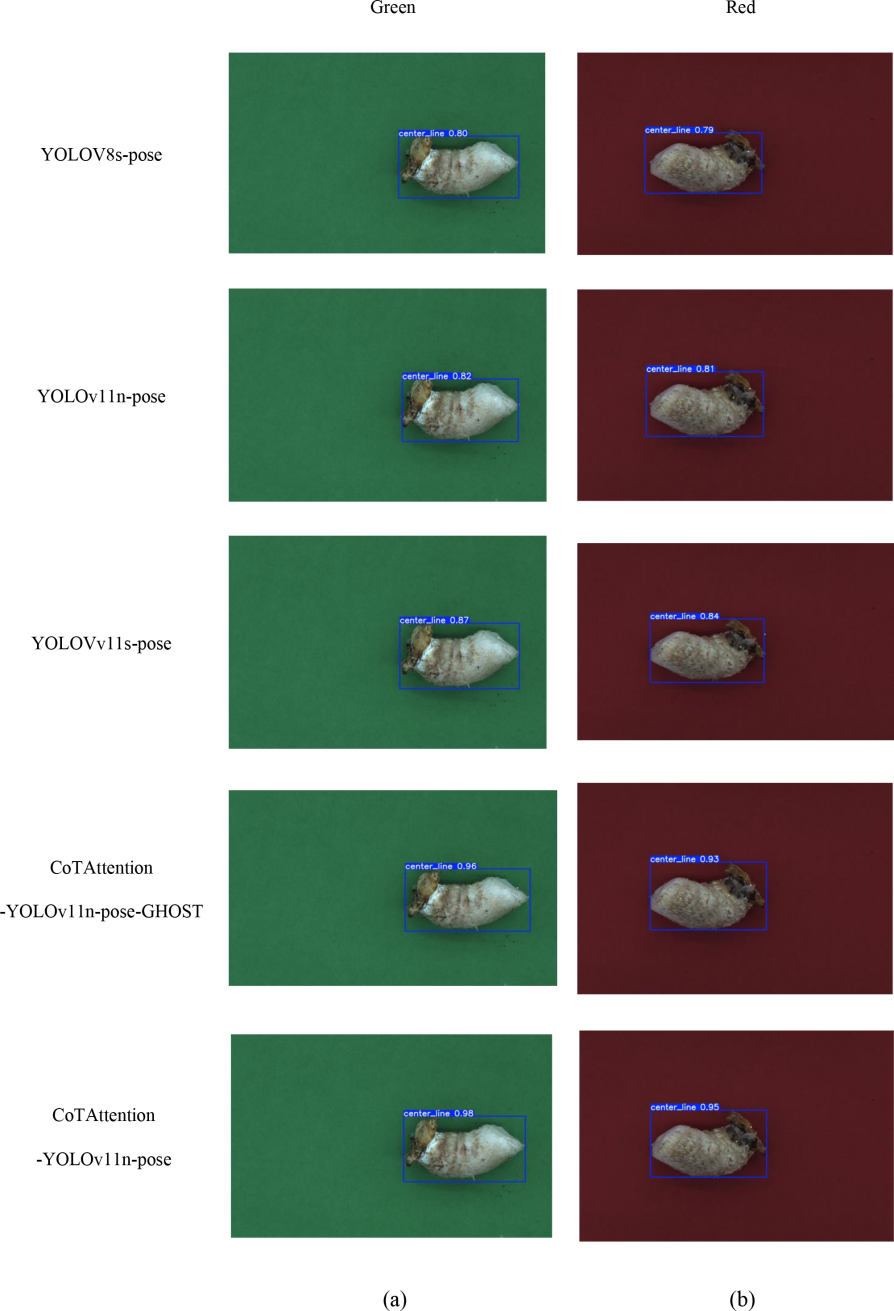
Graph of detection results of different centerline detection algorithms **(a)** Green-shiitake mushroom stipes **(b)** Red-shiitake mushroom stipes.

As shown in **Figure 11**, the models exhibit varying confidence levels across the two backgrounds. Specifically, YOLOv8s-pose achieves a confidence level of 0.80 on the green background and 0.79 on the red background, with only a 0.01 difference. This demonstrates the model’s stable confidence across different backgrounds, although its overall accuracy remains relatively low. Similarly, YOLOv11n-pose shows confidence levels of 0.82 and 0.81 on the green and red backgrounds, respectively, with minimal fluctuation, indicating consistent performance under varying background conditions. In contrast, YOLOv11s-pose reaches 0.87 on the green background and 0.84 on the red background, showing a slightly larger difference of 0.03. This suggests a more noticeable influence of background color on its confidence compared to the previous two models. Notably, CoTAttention-YOLOv11n-pose-GHOST achieves confidence levels of 0.96 and 0.93 on the green and red backgrounds, respectively. Despite the drop, it maintains a high confidence level while reducing model complexity. This performance can be attributed to the GHOST module, which enables lightweight feature processing, and the CoTAttention mechanism, which enhances feature interactions, thereby improving confidence across different color backgrounds. Finally, CoTAttention-YOLOv11n-pose achieves the highest confidence levels—0.98 on the green background and 0.95 on the red background—with a difference of 0.03. Although slightly affected by background color, its overall confidence remains high, demonstrating that the introduced attention mechanism significantly improves model confidence and highlighting its sensitivity to background variations. These results clearly indicate that model confidence in detection outcomes is jointly influenced by both architectural design and background characteristics.

**Table 5** presents the performance of various algorithms in detecting the centerline of shiitake mushroom stipes on both green and red backgrounds. In the green shiitake mushroom stipes detection task, different base models exhibit distinct performance characteristics. YOLOv8s-pose has high computational and parameter complexity, achieving a recall rate of 0.806, an average precision of 0.782, and an inference speed of 63.51ms, indicating considerable room for improvement in both performance and efficiency. YOLOv11n-pose features relatively lower computational and parameter scales, with a recall rate of 0.824, an average precision of 0.793, and an inference speed of 69.82ms, showing better balance between precision and efficiency than YOLOv8s-pose, though further optimization remains possible. The lightweight YOLOv11s-pose significantly reduces both model complexity and computational cost, while achieving a recall rate of 0.853, an average precision of 0.851, and an inference speed of 21.09ms, demonstrating strong potential in balancing detection accuracy and speed. Among the improved models, CoTAttention-YOLOv11n-pose incorporates the attention mechanism, leading to a substantial performance boost: the recall rate increases to 0.969 and the average precision reaches 0.978. Although computational cost and parameter count rise slightly, the inference speed remains at 25.63ms, indicating that enhanced feature extraction significantly improves detection accuracy. Further optimization is achieved in CoTAttention-YOLOv11n-pose-GHOST by integrating the GHOST module, which slightly reduces computational load and parameter size while maintaining a recall rate of 0.961 and an average precision of 0.972. The inference speed improves to 22.09ms, demonstrating a synergistic optimization of both model accuracy and efficiency.

**Table 5:** Performance of different stipe centerline detection algorithms.

Models	Epoch	Train on	Recall	AP	GFLOPs	Parameters	Speed_PyTorch(ms)
YOLOV8s-pose	100	Shiitake mushroom stipes(Green)	0.806	0.782	29.64	11423552	63.51
YOLOV11n-pose	100	Shiitake mushroom stipes(Green)	0.824	0.793	22.54	2662416	69.82
YOLOV11s-pose	100	Shiitake mushroom stipes(Green)	0.853	0.851	6.69	9715144	21.09
CoTAttention -YOLOV11n-pose	100	Shiitake mushroom stipes(Green)	0.969	0.978	14.9	4749432	25.63
CoTAttention -YOLOV11n-pose-GHOST	100	Shiitake mushroom stipes(Green)	0.961	0.972	14.2	4670096	22.09
YOLOV8s-pose	100	Shiitake mushroom stipes(Red)	0.758	0.766	29.64	11423552	67.41
YOLOV11n-pose	100	Shiitake mushroom stipes(Red)	0.796	0.775	22.54	2662416	70.13
YOLOV11s-pose	100	Shiitake mushroom stipes(Red)	0.838	0.812	6.69	9715144	23.97
CoTAttention -YOLOV11n-pose	100	Shiitake mushroom stipes(Red)	0.951	0.961	14.9	4749432	26.18
CoTAttention-YOLOV11n-Ghost-pose	100	Shiitake mushroom stipes(Red)	0.945	0.951	14.2	4670096	24.07

In the red background scenario for shiitake mushroom stipes detection, the performance of baseline models is significantly affected by background interference. YOLOv8s-pose achieves a recall rate of 0.758, an average precision of 0.766, and an inference speed of 67.41ms, showing a clear decline in accuracy compared to its performance in green backgrounds. YOLOv11n-pose demonstrates improved performance with a recall rate of 0.796, an average precision of 0.775, and an inference speed of 70.13ms, yet the gain in accuracy under red background conditions remains limited. YOLOv11s-pose preserves its lightweight advantage, achieving a recall rate of 0.838, an average precision of 0.812, and an inference speed of 23.97ms, indicating stronger adaptability to varying background conditions. Among the enhanced models, CoTAttention-YOLOv11n-pose incorporates an attention mechanism to improve feature focusing capability, achieving a recall rate of 0.951, an average precision of 0.961, and an inference speed of 26.18ms, which significantly enhances detection robustness. CoTAttention-YOLOv11n-Ghost-pose further improves performance with a recall rate of 0.945, an average precision of 0.951, and an inference speed of 24.07ms. By integrating the GHOST module and optimizing detection strategies, it effectively enhances performance in red background conditions, offering a more effective solution for object detection in complex background environments.

In summary, the improved CoTAttention fusion model demonstrates a superior balance between detection accuracy and computational efficiency in both green and red shiitake mushroom stipes detection scenarios. CoTAttention-YOLOv11n-Ghost-pose is selected as the centerline detection model due to its effective trade-off between precision and inference speed.

### 3.2 Error analysis of phenotypic assay

**Figure 12:**
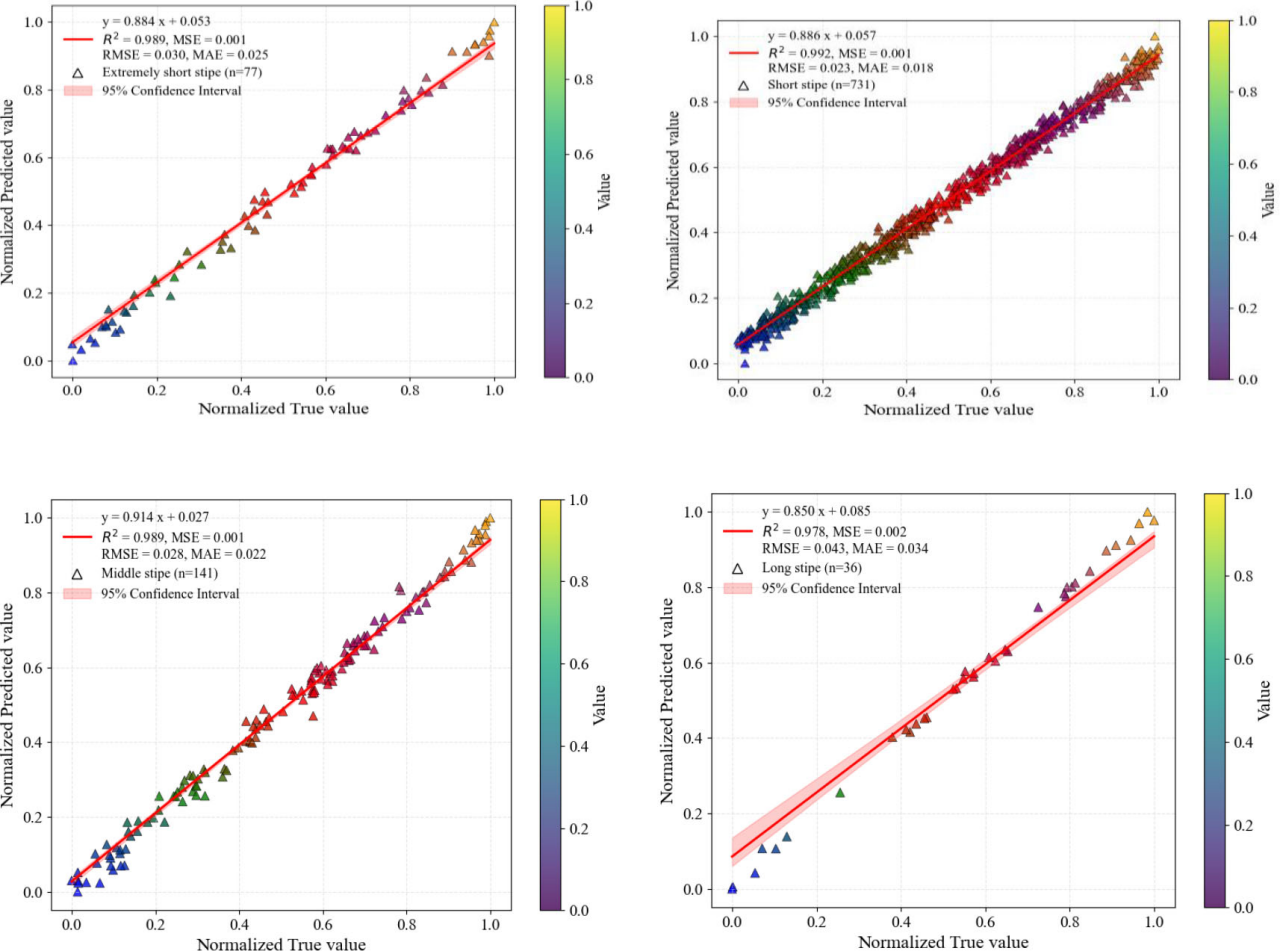
Predicted versus true values for different lengths of mycelium stipes.

Due to the special characteristics of stipe measurement, we used the length of the outer rectangle of the stipes as the true value and analyzed the correlation between the predicted and true values across four stipe length categories of shiitake mushroom—extremely short, short, Middle, and long—using Ridge regression. The results are presented in **Figure 12**. **Table 6** provides a more intuitive representation of the error distribution across different sample groups. The Short stipe group (sample size:731) demonstrated the best fit, with a correlation coefficient of 0.992 and an MSE of 0.001. This indicates that the model performs well in capturing small-scale stipe phenotypes when the sample size is sufficient. For the extremely short stipe group, the MAE increased to 0.025 due to the smaller sample size. Similarly, for the Long stipe group (sample size:36), which constitutes only 5% of the Short stipe group, the correlation coefficient decreased to 0.978, MSE increased to 0.002, and both RMSE (0.043) and MAE (0.034) were higher than those of other groups. Additionally, the dispersion of scattered points significantly increased, suggesting a decrease in model robustness to extreme values for longer stipes, likely due to the limited sample size and increased noise interference. The Middle stipe group (sample size:141) showed a correlation of 0.989 with an MSE of 0.001, while RMSE and MAE were 0.028 and 0.022, respectively. These results suggest that the growth patterns of Middle stipes may involve more complex nonlinear characteristics, or that the sample distribution is less uniform, which affects the model’s fitting performance.

**Table 6:** Plot of predicted versus true values for different lengths of stipe.

Metric	Extremely short(mm)	Short(mm)	Middle(mm)	Long(mm)
mumber	77	731	141	36
MAE	0.025	0.018	0.022	0.034
MSE	0.001	0.001	0.001	0.002
RMSE	0.030	0.023	0.028	0.043

The experimental results show that the algorithmic model proposed in this study maintains a low error level for all four lengths of shiitake mushroom stipes phenotypic detection, which can provide practical support for stipe phenotypic measurement in shiitake mushroom breeding.

**Table 7** shows the analysis of the phenotypic characteristics of shiitake mushroom stipes. We calculated the key phenotypic measurements of 985 shiitake mushroom stipes samples, and the data situation of each phenotype is shown in **Table 7**. By quantitatively analyzing the measured phenotypic characteristics, differences between different phenotypic categories can be explored. Several key phenotypic metrics are listed in **Table 7**, including the length and width, roundness, area, perimeter, long and short axes of the outer tangent rectangle of the shiitake stipe, as well as the mean values of the color channels R, G, and B, and the mean values of the grayscales. The statistics of these metrics cover the maximum and minimum values of mean, maximum, minimum, standard deviation, and standard score. In the statistical analysis of the phenotypic features of the shiitake mushroom stipes, it was found that the mean values of the morphological features such as centerline length, outer rectangle height, and outer rectangle length were 67.18 mm, 30.59 mm, and 56.29 mm, respectively, which reflected the basic dimensional features of the shiitake mushroom stipes. The maximum and minimum values of these features reveal the range of variability of the data, while the standard deviation measures the degree of dispersion of the data - for example, the standard deviation of the centerline length is 10.11mm, indicating that there is a certain degree of variability in the data. The analysis of color characteristics (R, G, and B means) and grayscale means reflected the visual characteristics of shiitake mushroom stipes: the R channel mean was the highest (107.48), the grayscale mean was 98.17, and the standard deviation of these metrics was relatively small, suggesting that shiitake mushroom Shiitake mushroom stipe had a more consistent color distribution. The mean value of curvature was 0.20, indicating a certain degree of curvature. These statistical data comprehensively presented the phenotypic information of shiitake mushroom stipes, which provided the basis for the in-depth study of their growth characteristics.

**Table 7:** Analysis of measurements of phenotypic characteristics of shiitake mushroom stipes.

Phenotype category	Mean value(mm)	Maximum value(mm)	Minimum value(mm)	Standard deviation(mm)	Median value(mm)
Centerline	67.18	84.05	47.98	10.11	67.53
Outer rectangular width	30.59	35.68	25.89	2.79	31.06
Exterior rectangular length	56.29	70.32	39.69	8.24	56.47
Maximum thickness	25.71	31.3	21.7	2.37	26.33
Minimum thickness	24.03	30.69	20.2	2.76	23.28
Value Average thickness	24.9	31.46	20.96	2.48	24.33
Curvature	0.20	0.33	0.12	0.07	0.20
Weight	1.60	4.06	0.76	0.81	1.48
Area	1203.21	1731.75	950.8	179.71	1180.68
Perimeter	167.03	220.62	133.67	20.40	161.66
R	107.48	120.59	78.67	9.00	109.14
G	100.12	116.9	67.9	10.57	102.51
B	86.90	100.56	60.77	8.90	88.89
Gray mean	98.17	112.68	69.11	9.46	100.07

## 4 Discussion

In this study, a novel algorithm for shiitake mushroom stipes centerline detection was proposed. To evaluate the performance of different models in the centerline detection task, two background environments—green and red—were constructed, and five pose estimation models were selected for comparative experiments: YOLOv8s-pose, YOLOv11n-pose, YOLOv11s-pose, CoTAttention-YOLOv11n-Ghost-pose, and CoTAttention-YOLOv11n-pose. The experimental results were visualized in detection boxes labeled with the “center_line” confidence score. From the perspective of confidence levels, the models exhibited varying performance in the two backgrounds due to differences in network architecture. Taking CoTAttention-YOLOv11n-pose as an example, its confidence level reached 0.98 on the green background, nearly the upper limit of confidence, indicating that the improved model can accurately extract and match key centerline features with the support of the feature attention mechanism, particularly under conditions where the green background provides strong contrast with the stipe, thereby enhancing detection confidence. However, when the background was switched to red, the confidence level decreased to 0.95, which remains within the high-confidence range. This drop is attributed to the red background being more similar in color to the stipe, leading to feature confusion and increased difficulty in distinguishing features, which in turn slightly reduced the model’s confidence. Similarly, YOLOv8s-pose achieved confidence levels of 0.80 on the green background and 0.79 on the red background, while YOLOv11n-pose scored 0.82 and 0.81, respectively. The small fluctuations in confidence levels across models reflect the influence of background color on feature identification and confidence estimation, a finding consistent with the results reported by Magalhães et al. (2021). From the perspective of red and green backgrounds, the green background, due to its appropriate contrast with the color of the stipe, is more conducive to the extraction of key features such as the stipe contour and center line by most models (e.g., CoTAttention-YOLOv11n-pose, YOLOv11n-pose, etc.). This reduces background noise interference and leads to more accurate feature recognition and matching, thereby enhancing the model’s confidence in the detection results. As a result, most models exhibit relatively higher confidence levels on the green background. In contrast, the red background tends to cause feature confusion with the stipe color tone, increasing the difficulty of feature discrimination. Even when the model successfully detects the center line, the confidence level is generally lower, highlighting the strong interference effect of the red background on the model’s feature recognition capability.

In terms of prediction error, the performance of the Ridge regression model for mushroom stipes length prediction in this study is primarily constrained by sample size: the short group (n=731) achieved a high goodness of fit of 0.992 and low errors with sufficient samples, confirming the error dilution and variation coverage effects of large samples, which is consistent with the conclusions of Dong et al. (2025). Accuracy declines sharply with reduced sample size: the correlation coefficient of the middle group (n=141) drops to 0.989, while the long group (n=36, accounting for less than 5% of the total sample size) shows doubled MSE and a surge in errors. Critically, insufficient sample size is fundamental: as a key extreme phenotype for breeding, the scarce samples of the long-length group both amplify environmental noise and impair model robustness. Additionally, existing studies have not quantified the critical sample size or disentangled the “sample size-phenotypic variation” interaction effect. In summary, the model is overly dependent on sample size and only has practical value for groups with sufficient samples. Future work should expand the sample size of medium- and long-length extreme phenotypes and integrate data augmentation techniques to enhance model generalization for breeding scenarios.

In summary, both the model structure and background color have a joint impact on the confidence level of stipe centerline detection. The green background is generally more favorable for improving detection confidence, whereas the red background tends to reduce confidence due to feature confusion. Future research can focus on background-adaptive feature enhancement—for example, by designing dynamically adjustable feature extraction modules to better cope with background interference—and on optimizing attention mechanisms to strengthen the model’s focus on key stipe features while reducing the influence of background noise. These improvements can enhance the model’s confidence and robustness in complex environments, thereby providing more reliable support for accurate stipe phenotypic detection.

## 5 Conclusions

In the breeding process of shiitake mushroom, the key phenotypic traits of the stipe have the problem of not being able to be measured or not being measured manually with high accuracy, which seriously hinders the improvement of breeding efficiency and quality improvement. To solve this technical problem, this study proposes a detection method that integrates image processing techniques: firstly, an ACmix-ADown-YOLOv11n stipe detection model is established, which achieves an average precision (AP) of 93.7% and a detection speed of 23.97 ms; the detection frame is input into an EfficientSAM network to obtain a stipe segmentation map, and then, stipe edge information derived from deep learning is employed to calculate 12 key phenotypic traits. which is the most important in the mushroom breeding process. The image processing technology based on OpenCV was used to calculate 12 key phenotypic features; at the same time, the CoTAttention-YOLOv11n-Ghost-pose algorithm was used to predict the centerline of the stipe, with an AP of up to 0.961, and the detection speed of 22.09 ms. The experimental results showed that the R²s of the predicted values of the extremely short, short, middle, and long stipe to the real value were 0.989, 0.992, 0.989, 0.978, RMSE 0.030, 0.023, 0.028, 0.043 respectively, which fully verified the effectiveness of the method. In summary, the detection method proposed in this study provides a powerful guidance and effective solution for the accurate prediction of key traits of shiitake mushroom stipes, which is of great practical significance and application value for promoting the intelligent breeding process and improving the breeding quality of shiitake mushroom.

In future work, research will be deepened and expanded along three key dimensions. First, multispectral imaging technology will be introduced to integrate hyperspectral data with RGB images, enabling the construction of a multidimensional feature space that incorporates both phenotypic traits and nutritional information. Second, machine learning algorithms will be applied to quantitatively analyze the influence pathways of various factors on shiitake mushroom phenotypes. Third, open data collection platforms will be developed to incorporate sample data from diverse cultivation conditions and growth stages, thereby enhancing model generalizability through transfer learning. These improvements are expected to transcend current research limitations and promote the industrial application of intelligent phenotypic detection technologies for shiitake mushroom stipes.

## Data Availability

The original contributions presented in the study are included in the article/supplementary material. Further inquiries can be directed to the corresponding authors.

## References

[B1] AS R. A. Gopalan S. (2022). Comparative analysis of eight direction sobel edge detection algorithm for brain tumor MRI images. Proc. Comput. Sci. 201, 487–494. doi: 10.1016/j.procs.2022.03.063

[B2] Chen B. Gu S. Huang G. Lu X. Chang W. Wang G. . (2025). Improved estimation of nitrogen use efficiency in maize from the fusion of UAV multispectral imagery and LiDAR point cloud. Eur. J. Agron. 168, 127666. doi: 10.1016/j.eja.2025.127666

[B3] da Silva Milhorini S. Simas F. F. Smiderle F. R. de Jesus L. I. Rosado F. R. Longoria E. L. . (2022). β-Glucans from the giant mushroom Macrocybe titans: Chemical characterization and rheological properties. Food Hydrocolloids. 125, 107392. doi: 10.1016/j.foodhyd.2021.107392. ISSN 0268-005X.

[B4] Demirel S. Çelikten A. Akpulat A. Demir M. K. Bingöl E. Gületkin İ. . (2025). Real-time polyp detection: A speed and performance analysis of YOLOv5 and YOLOv6. Osmaniye Korkut AtaÜniversitesi Fen Bilimleri EnstitüsüDergisi. 8, 1240–1257. doi: 10.47495/okufbed.1544536

[B5] Deng L. Miao Z. Zhao X. Yang S. Gao Y. Zhai C. . (2025). HAD-YOLO: an accurate and effective weed detection model based on improved YOLOV5 network. Agronomy. 15, 57. doi: 10.3390/agronomy15010057

[B6] Di J. Xi K. Yang Y. (2025). An enhanced YOLOv8 model for accurate detection of solid floating waste. Sci. Rep. 15, 25015. doi: 10.1038/s41598-025-10163-2, PMID: 40646062 PMC12254491

[B7] Dong S. L. Cao L. Liu W. J. Huang M. Sun Y. X. Zhang Y. Y. . (2025). System-specific aquaculture annual growth rates can mitigate the trilemma of production, pollution and carbon dioxide emissions in China. Nat. Food. 6, 365–374. doi: 10.1038/s43016-025-01122-1, PMID: 39934256

[B8] Elharrouss O. Hmamouche Y. Kamal Idrissi A. El Khamlichi B. El Fallah-Segrouchni A. (2023). Refined edge detection with cascaded and high-resolution convolutional network. Pattern Recognition. 138, 109361. doi: 10.1016/j.patcog.2023.109361

[B9] Gui H. Su T. Jiang X. Li L. Xiong L. Zhou J. . (2025). FS-YOLOv9:a frequency and spatial feature-based YOLOv9 for real-time breast cancer detection. Acad. Radiol. 32, 1228–1240. doi: 10.1016/j.acra.2024.09.048, PMID: 39406579

[B10] Hao J. Yan G. Wang L. Pei H. Xiao X. Zhang B. . (2025). Lightweight transmission line outbreak target obstacle detection incorporating ACmix. Processes. 13, 271. doi: 10.3390/pr13010271

[B11] He L. Zhou Y. Liu L. Cao W. Ma J. (2025). Research on object detection and recognition in remote sensing images based on YOLOv11. Sci. Rep. 15, 14032. doi: 10.1038/s41598-025-96314-x, PMID: 40269047 PMC12019343

[B12] Ji D. Liu Y. Wang C. (2022). “ Research on image edge detection based on improved canny operator,” in 2022 3rd International Conference on Information Science, Parallel and Distributed Systems (ISPDS). (Guangzhou, China: IEEE) 229–232.

[B13] Ji S. Granato D. Wang K. Hao S. Xuan H. (2025). Detecting the authenticity of two monofloral honeys based on the canny-GoogLeNet deep learning network combined with three-dimensional fluorescence spectroscopy. Food Chem. 485, 144509. doi: 10.1016/j.foodchem.2025.144509, PMID: 40306056

[B14] Jindal R. Mittal S. K. (2025). “ Artificial intelligence and machine vision-based assessment of rice seed quality,” in Applied Data Science and Smart Systems (Boca Raton, USA: CRC Press), 603–609.

[B15] Liao L. Song C. Wu S. Fu J. (2025). A novel YOLOv10-based algorithm for accurate steel surface defect detection. Sensors. 25, 769. doi: 10.3390/s25030769, PMID: 39943407 PMC11820220

[B16] Liu C. Chen R. Chen K. Xu J. (2022). Ellipse detection using the edges extracted by deep learning. Mach. Vision Appl. 33, 63. doi: 10.1007/s00138-022-01319-5

[B17] Liu S. R. Ke B. R. Zhang W. R. Liu X. R. Wu X. P. (2017). Breeding of new Ganoderma lucidum strains simultaneously rich in polysaccharides and triterpenes by mating basidiospore-derived monokaryons of two commercial cultivars. Scientia Hortic. 216, 58–65. doi: 10.1016/j.scienta.2016.12.016

[B18] Ma W. Qi X. Sun Y. Gao R. Ding L. Wang R. . (2024). Computer vision-based measurement techniques for livestock body dimension and weight:A review. Agriculture. 14, 306. doi: 10.3390/agriculture14020306

[B19] Magalhães S. A. Castro L. Moreira G. dos Santos F. N. Cunha M. Dias J. . (2021). Evaluating the single-shot multibox detector and YOLO deep learning models for the detection of tomatoes in a greenhouse. Sensors. 21, 3569. doi: 10.3390/s21103569, PMID: 34065568 PMC8160895

[B20] Mesele E. Yaekob A. T. Zeslassie A. (2024). Valuation of the growth response of oyster(Pleurotus ostreatus)mushroom on partially composted sesame stalk with different blends of wheat straw. Discover Food. 4, 80. doi: 10.1007/s44187-024-00147-y

[B21] Miraldo A. Sundh J. Iwaszkiewicz-Eggebrecht E. Buczek M. Goodsell R. Johansson H. . (2025). Data of the Insect Biome Atlas: a metabarcoding survey of the terrestrial arthropods of Sweden and Madagascar. Sci. Data. 12, 835. doi: 10.1038/s41597-025-05151-0, PMID: 40399316 PMC12095508

[B22] Qi K. Yang Z. Fan Y. Song H. Liang Z. Wang S. . (2025). Detection and classification of Shiitake mushroom fruiting bodies based on Mamba YOLO. Sci. Rep. 15, 15214. doi: 10.1038/s41598-025-00133-z, PMID: 40307284 PMC12043908

[B23] Ran X. Liu Y. Pan H. Y. Wang J. Duan Q. (2025). Shrimp phenotypic data extraction and growth abnormality identification method based on instance segmentation. Comput. Electron. Agric. 229, 109701. doi: 10.1016/j.compag.2024.109701

[B24] Sahu C. K. Pattnayak S. B. Behera S. Mohanty M. R. (2020). “ A comparative analysis of deep learning approach for automatic number plate recognition,” in 2020 Fourth International Conference on I-SMAC (IoT in Social, Mobile, Analytics and Cloud) (I-SMAC). (Palladam, India: IEEE) 932–937.

[B25] Verma A. Dhanda N. Yadav V. (2023). Binary particle swarm optimization based edge detection under weighted image sharpening filter. Int. J. Inf. Technol. 15, 289–299. doi: 10.1007/s41870-022-01127-0

[B26] Wang C. Zhou Y. Weng Z. Wei N. (2025). A novel functional reconstruction method between rail RCF crack parameters and ACFM feature. Construction Building Materials. 483, 141636. doi: 10.1016/j.conbuildmat.2025.141636

[B27] Wu F. Yang Z. Mo X. Wu Z. Tang W. Duan J. . (2023). Detection and counting of banana bunches by integrating deep learning and classic image-processing algorithms. Comput. Electron. Agric. 209, 107827. doi: 10.1016/j.compag.2023.107827

[B28] Xiang Q. Adil B. Chen Q. Gu Y. Zeng X. Li X. . (2021). “ Shiitake mushroom (Lentinula edodes (Berk.) sing.) breeding in China,” in Advances in Plant Breeding Strategies: Vegetable Crops: Volume 10: Leaves, Flowerheads, Green Pods, Mushrooms and Truffles ( Springer International Publishing, Cham), 443–476.

[B29] Xiong Y. Varadarajan B. Wu L. Xiang X. Xiao F. Zhu C. . (2024). “ Efficientsam: Leveraged masked image pretraining for efficient segment anything,” in Proceedings of the IEEE/CVF Conference on Computer Vision and Pattern Recognition. (Seattle, WA, USA: IEEE/CVF) 16111–16121.

[B30] Xu H. Fu L. Li J. Lin X. Chen L. Zhong F. . (2024). A method for analyzing the phenotypes of nonheading Chinese cabbage leaves based on deep learning and openCV phenotype extraction. Agronomy. 14, 699. doi: 10.3390/agronomy14040699

[B31] Xu X. Li J. Zhou J. Feng P. Yu H. Ma Y. . (2025). Three-dimensional reconstruction, Phenotypic traits extraction, and yield estimation of shiitake mushrooms based on structure from motion and multi-view stereo. Agriculture. 15, 298. doi: 10.3390/agriculture15030298

[B32] Yang W. Chen X. D. Wang H. Mao X. (2024). Edge detection using multi-scale closest neighbor operator and grid partition. Visual Comput. 40, 1947–1964. doi: 10.1007/s00371-023-02894-y

[B33] Yang H. Fang C. (2025). Semantic edge detection of bipolar plate welding area based on EDFormer. Comput. Integrated Manufacturing System. 31, 1569. doi: 10.13196/j.cims.2024.0177

[B34] Yang D. Yang H. Liu D. . (2024). Research on automatic 3D reconstruction of plant phenotype based on Multi-View images. Comput. Electron. Agric. 220, 108866. doi: 10.1016/j.compag.2024.108866

[B35] Zhang W. Jiang F. (2025). AHN-YOLO:A lightweight tomato detection method for dense small-sized features based on YOLO architecture. Horticulturae. 11, 639. doi: 10.3390/horticulturae11060639

[B36] Zhang Y. Sheng J. Wang Z. Meng Z. Sun Y. (2025). Dynamic modeling and improved PiDiNet edge detection of multi-process mixed braiding net. Measurement. 240, 115566. doi: 10.1016/j.measurement.2024.115566

[B37] Zheng X. Gong W. Li C. Zhang L. Bian Y. Kwan H. . (2019). Comprehensive evaluation of shiitake strains (Lentinus edodes, Agaricomycetes) based on polysaccharide content and agronomic traits. Int. J. Medicinal Mushrooms. 21, 851–864. doi: 10.1615/IntJMedMushrooms.2019031913, PMID: 32450025

[B38] Zou L. Zheng Y. Lu J. (2024). “ An edge detection method for welding pool based on an improved canny algorithm,” in Journal of Physics: Conference Series, (Changsha, China: IOP Publishing) Vol. 2785, 012013.

